# Effective Gene Expression Prediction and Optimization from Protein Sequences

**DOI:** 10.1002/advs.202407664

**Published:** 2025-01-09

**Authors:** Tuoyu Liu, Yiyang Zhang, Yanjun Li, Guoshun Xu, Han Gao, Pengtao Wang, Tao Tu, Huiying Luo, Ningfeng Wu, Bin Yao, Bo Liu, Feifei Guan, Huoqing Huang, Jian Tian

**Affiliations:** ^1^ State Key Laboratory of Animal Nutrition and Feeding Institute of Animal Sciences Chinese Academy of Agricultural Sciences Beijing 100193 China; ^2^ National Key Laboratory of Agricultural Microbiology Biotechnology Research Institute Chinese Academy of Agricultural Sciences Beijing 100081 China; ^3^ School of Life Sciences Tianjin University Tianjin 300110 China

**Keywords:** amino acid expression index, mutant generation, predicting protein expression, soluble expression, transfer learning

## Abstract

High soluble protein expression in heterologous hosts is crucial for various research and applications. Despite considerable research on the impact of codon usage on expression levels, the relationship between protein sequence and expression is often overlooked. In this study, a novel connection between protein expression and sequence is uncovered, leading to the development of SRAB (Strength of Relative Amino Acid Bias) based on AEI (Amino Acid Expression Index). The AEI served as an objective measure of this correlation, with higher AEI values enhancing soluble expression. Subsequently, the pre‐trained protein model MP‐TRANS (MindSpore Protein Transformer) is developed and fine‐tuned using transfer learning techniques to create 88 prediction models (MPB‐EXP) for predicting heterologous expression levels across 88 species. This approach achieved an average accuracy of 0.78, surpassing conventional machine learning methods. Additionally, a mutant generation model, MPB‐MUT, is devised and utilized to enhance expression levels in specific hosts. Experimental validation demonstrated that the top 3 mutants of xylanase (previously not expressed in *Escherichia coli*) successfully achieved high‐level soluble expression in *E. coli*. These findings highlight the efficacy of the developed model in predicting and optimizing gene expression based on protein sequences.

## Graphical abstract

Understanding the correlation between protein sequences and expression is essential for many research and applications. This study introduced a new mathematical model to confirm the association between protein sequence and expression. The pre‐trained protein model, MP‐TRANS, is developed and fine‐tuned using transfer learning techniques to create prediction models, as well as models for generating mutants to adjust soluble expression levels.

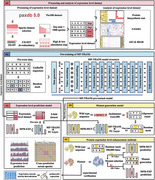

## 1 Introduction

The efficient soluble expression of specific proteins in cells is a prerequisite for functional research and practical applications in all areas of the life sciences. Numerous factors have been identified that clearly influence soluble protein expression, including protein structure,^[^ ^1^ ^]^ codon selection,^[^ ^2^ ^]^ transcriptional and translational efficiency,^[^ ^3^ ^]^ mRNA structure and stability,^[^ ^4^ ^]^ etc. However, less than 50% of bacterial proteins and less than 15% of nonbacterial proteins can be successfully expressed in soluble form in *Escherichia coli*.^[^ ^5^ ^]^ Achieving high levels of protein expression remains a key challenge for research and practical applications.

Conventional methods of increasing soluble expression levels involve changing expression hosts, using various expression vectors,^[^ ^6^ ^]^ and fusing the target protein with solubility tags^[^ ^7^ ^]^ or molecular chaperones.^[^ ^8^ ^]^ From the perspective of the amino acid sequence itself, current research has focused on rational design and altering specific amino acids or particular fragments of protein sequences to elucidate their impact on protein expression. For example, Shinoda et al.^[^ ^9^ ^]^ established the “alpha‐helix rule” and the “hydropathy contradiction rule” to select residues that improve heterologous recombinant protein production. Hu et al.^[^ ^10^ ^]^ replaced digestive sites on the surfaces of pepsin and trypsin with polar amino acids, then introduced hydrophobic amino acids to reshape the catalytic pocket, thus increasing the soluble expression levels by 45‐fold. It is clear from these 2 studies that appropriate methods rely heavily on the experience and intuition of the researchers involved, with the best solutions identified through trial and error; only certain aspects or factors can be optimized.

Artificial intelligence (AI)‐based methods typically start at the codon level, combining codon selection with deep learning models to extract new features from input data, revealing why codon selection affects high soluble expression levels.^[^ ^2^, ^11^ ^]^ Additionally, expression‐level prediction models trained using features such as host‐specific codon preference, codon recognition number, codon count, and minimum free energy of folding can achieve good generalizability.^[^ ^12^ ^]^ Therefore, we hypothesized that the identification of soluble expression‐related features from expressed sequence fragments that differentiate soluble expressing proteins can be achieved with deep learning models.^[^ ^13^ ^]^

The advent of rapid sequencing technologies has led to the generation of vast quantities of protein sequence data, enabling the construction of large models based on the amino acid sequences of proteins. Transfer learning technology within deep learning has shown unprecedented performance in several protein engineering computational tasks, especially in terms of protein representation. For example, ProteinBERT^[^ ^14^ ^]^ outperforms previous models on multiple benchmarks covering a wide range of protein characteristics, whereas ProGen^[^ ^15^ ^]^ generates functional protein sequences across different families through a pretrained large language model. This progress suggests that it is possible for deep learning models to learn complex biological systems, assess the long‐distance connections of proteins, and thus extract patterns and relationships inherent in protein sequences.^[^ ^16^ ^]^

Here, through in‐depth analysis of large amounts of protein data, we first identified key factors contributing to protein soluble expression, such as physicochemical properties and the biosynthetic costs of amino acids. Next, we proposed a new mathematical model, SRAB (Strength of Relative Amino Acid Bias) based on AEI (Amino Acid Expression Index), to quantify and predict protein expression levels from protein sequences. Then, we developed a series of deep learning models that use transfer learning to explore the complex features hidden in protein sequences closely related to their soluble expression levels; this resulted in 88 soluble expression level prediction models (MPB‐EXPs) for 88 species, as well as a mutant generation model (MPB‐MUT) for the modification of soluble expression levels. We have completed the prediction and enhancement of soluble expression of the target protein in 88 species solely from the host and protein sequence perspectives.

## 2 Results

### 2.1 Overview of our Work on Enhancing Protein Expression Levels

**Figure 1:**
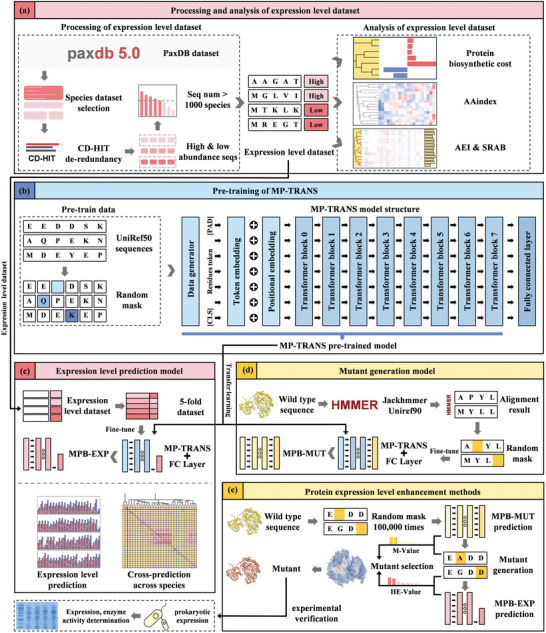
Workflow to predict and generate mutants to enhance soluble expression of proteins. a) Construction, processing, and analysis of the expression level data set. b) Pretraining of the MP‐TRANS model. Using the transfer learning method, MP‐TRANS was fine‐tuned to yield the c) protein expression level prediction model MPB‐EXP and d) mutant generation model MPB‐MUT. e) Design process for mutants that enhance protein expression level. Based on the results generated by MPB‐MUT combined with the predictions from MPB‐EXP, the solubility expression levels of specific proteins in the target host organisms can be enhanced.

In this study, a cutting‐edge transfer learning framework was developed to explore the intricate interplay between protein sequences and their soluble expression levels. This innovative framework enabled the accurate prediction of expression levels and the strategic engineering of mutants to enhance protein expression. The methodology involved the construction of a comprehensive protein expression data set derived from processed abundance information (**Figure** **1**a), followed by the development of the MP‐TRANS (MindSpore Protein Transformer) model incorporating Transformer layers for both pretraining and transfer learning (Figure 1b). This model was specially designed to learn generalized protein sequence features, ultimately enhancing the optimization of downstream tasks. Subsequently, fine‐tuning the MP‐TRANS model using the expression data set resulted in the development of 88 species‐specific MPB‐EXP models for precise expression level prediction (Figure 1c). To improve heterologous protein expression, mutants were designed by leveraging homologous sequences to train the mutant generation model. This model, MPB‐MUT, was fine‐tuned using MP‐TRANS and utilized to generate mutants, which were carefully selected based on MPB‐EXP predictions for experimental validation (Figure 1e).

### 2.2 Analysis of Sequence Factors that Affect Protein Expression Levels

To train the MPB‐EXP model, we constructed a protein expression data set using the PaxDB database and then evaluated the quality for model training. Raw data containing 170 species were downloaded from the PaxDB database.^[^ ^17^ ^]^ Then, the data was processed as described in Section 4.1 (Protein Expression Level Data Set Collection and Processing). Recognizing that these abundance levels differ among different species, we uniformly classified them into relative categories of high, medium, and low abundance. After data processing, we obtained 88 core data sets for 88 species, which were used in subsequent analysis and model training. Each protein entry included the protein ID, amino acid sequence, abundance value, and expression level (high or low).

**Figure 2:**
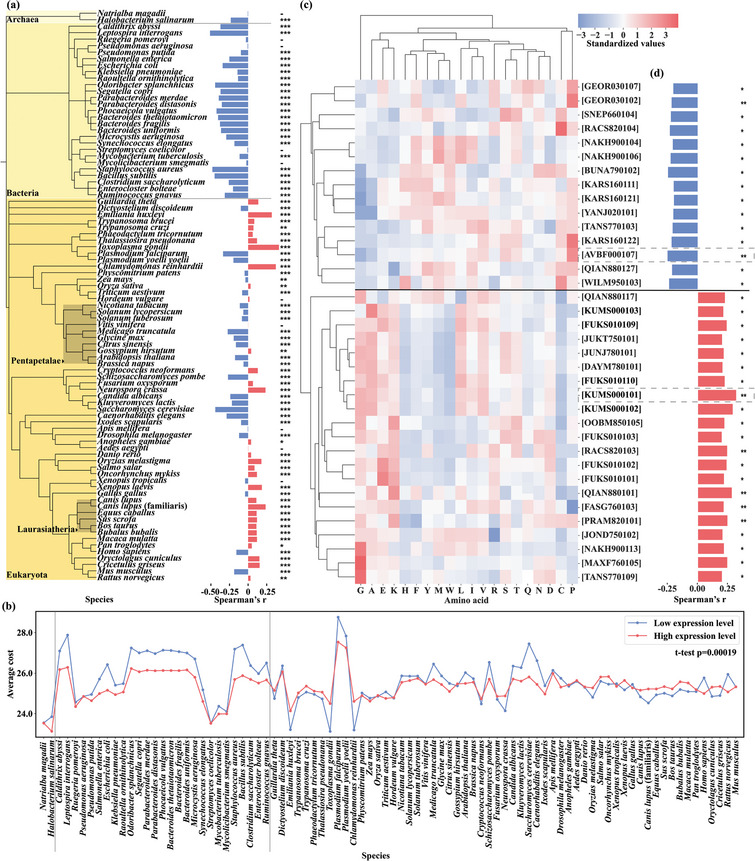
Relationships among protein expression levels, protein biosynthetic costs, and AAindex properties. a) Correlation between protein abundance and average biosynthetic cost in each species. b) Average biosynthetic costs of high‐ and low‐expression proteins in each species. c) Standardized values of 36 key amino acid properties and hierarchical clustering of these values at the characteristic and amino acid levels. d) Average correlation coefficients for 36 key properties with protein abundances of 88 species. The calculation involved computing the average value for each protein relative to each characteristic, calculating the correlation coefficient of this protein abundance in each species, and then calculating the average correlation coefficient of each characteristic across all 88 species. The 2 key properties indicated by the dotted lines are the property with the highest negative correlation (AVBF000107, Slopes tripeptide FDPB PARSE neutral, Spearman correlation coefficient = −0.25, *p* < 0.005) and the property with the highest positive correlation (KUMS000101, Distribution of amino acid residues in the 18 nonredundant families of thermophilic proteins, Spearman correlation coefficient = 0.32, *p* < 0.005). The figures show only the IDs of the properties. Detailed explanations and property values are outlined at https://www.genome.jp/aaindex. In the figure, asterisks are used to indicate the *p*‐Values associated with the Spearman correlation coefficient: “*” signifies *p* < 0.05, “**” denotes *p* < 0.01, and “***” represents *p* < 0.001, reflecting varying levels of statistical significance. The Protein Expression Level dataset contains 88 species, with a comprehensive sample size of 305774 protein sequences. Specifically, it includes 150 142 protein sequences characterized by high expression levels and 155 632 sequences by low expression levels.

Esley et al.^[^ ^18^ ^]^ reported that proteins with high soluble expression tend to include amino acids with lower biosynthetic cost, as determined by the number of high‐energy phosphate bonds (∼P). Here, we calculated the average biosynthetic cost per protein in each species and Spearman's correlation coefficient between the protein abundance and average biosynthetic costs. The results showed that proteins with high abundance exhibited a preference for amino acids with lower biosynthesis costs in most organisms (**Figure** **2**a). Especially in archaea and prokaryotes, protein abundances across all species showed negative correlations with the average biosynthetic cost of selected amino acids. The same negative correlation trend was observed in some eukaryotes, such as *Saccharomyces cerevisiae* and *Plasmodium falciparum*. Conversely, in some other eukaryotes, such as *Toxoplasma gondii* and *Chlamydomonas reinhardtii*, high‐abundance proteins tend to have relatively high levels of amino acids with high biosynthetic costs. Importantly, species that are evolutionarily related tend to have similar patterns of correlation between protein abundance and biosynthetic cost. For example, the superorder Laurasiatheria showed a positive correlation, whereas most species in the clade Pentapetalae showed a negative correlation (Figure 2a). We also compared the average protein synthesis costs between high‐ and low‐expression proteins in each species, as shown in Figure 2b. Highly expressed proteins showed lower average biosynthetic costs than proteins with low expression levels in all prokaryotes and archaea, except for *Pseudomonas aeruginosa*, which exhibited nearly identical costs for high‐ and low‐expression proteins. These observations confirmed that the average biosynthetic costs of amino acids in highly expressed proteins are relatively low.

Next, we identified 36 key properties per protein based on the amino acid property index values from the AAindex database (see Section 4.2 Expression level data analysis) and computed the average Spearman's correlation coefficient with protein abundance, the relevant information of these key properties is shown in Data S1 (Supporting Information). The results of hierarchical clustering showed that the properties of amino acids could be grouped into 2 child nodes at the root, which were positively and negatively correlated with expression level, respectively (Figure 2c,d). There were 15 negatively correlated properties and 21 positively correlated properties; all had correlation coefficients greater than 0.2 (Figure 2d). In the cluster exhibiting a positive correlation with expression level, the AAindex values of glycine (G), alanine (A), glutamate (E), and lysine (K) were higher, whereas those of cysteine (C) and proline (P) were lower. Conversely, the AAindex values of these amino acids were the opposite in the cluster exhibiting a negative correlation with expression level. The results showed that the properties most strongly associated with protein expression level were the amino acid conformational preference and solvation of polar backbone atoms in peptides and proteins (negatively correlated, ID = AVBF000107, Spearman correlation coefficient = −0.25, *P* < 0.01), as well as the distribution of amino acid residues in the 18 nonredundant families of thermophilic proteins (positively correlated, ID = KUMS000101, Spearman correlation coefficient = 0.32, *P* < 0.01). Among the selected properties, those positively correlated with protein soluble expression were mostly related to amino acid frequency, whereas those showing a negative correlation were largely associated with amino acid hydrophobicity. These properties can be used to identify potential features encoded inside the sequence with an effect on expression.

### 2.3 Mathematical Model of Protein Sequence to its Expression

**Figure 3:**
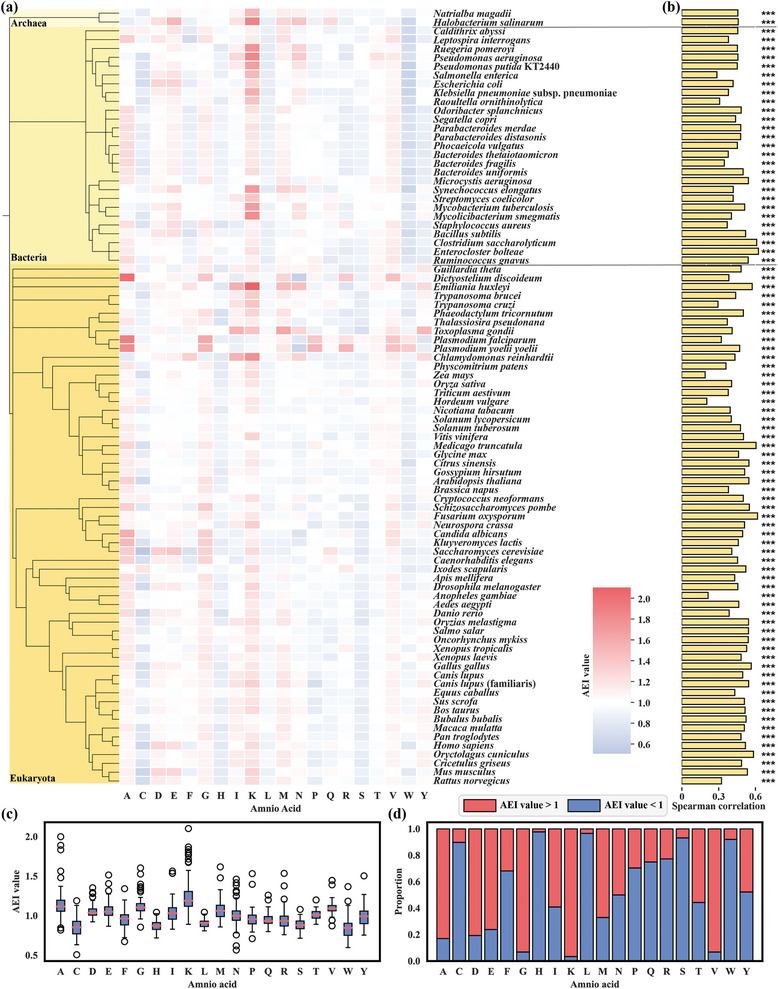
Quantifying the relationship between amino acid selection and soluble expression level. a) Heat map showing the AEI values for each species relative to the 20 amino acids. b) Correlation between protein abundance and SRAB values for all protein sequences from the selected PaxDB data set across 88 selected species. c) Distribution of AEI values for the 20 amino acids across different species. d) Comparison of the AEI values of the 20 amino acids to the value of 1 across different species. In the figure, asterisks are employed to signify the *P*‐Values corresponding to the Spearman correlation coefficient, where “*” stands for *p* < 0.05, “**” for *p* < 0.01, and “***” for *p* < 0.001, thereby indicating the statistical significance of the correlations.

We introduce AEI as a new metric for each amino acid for each species. We calculate a specific value, the AEI value (see Section 4.3 Amino acid and protein expression level connection), based on the protein abundance in the expression level dataset and the frequency of different amino acids in these proteins. The 20 AEI values for 20 amino acids corresponding to each of the 88 species can be used to quantify the relationship between amino acid selection and soluble expression levels. Detailed AEI value data is shown in Data S2 (Supporting Information). The AEI values for each species relative to the 20 amino acids are presented in a heat map in **Figure** **3**a; the overall data analysis is depicted in Figure 3c,d. The results showed that lysine (K), alanine (A), glycine (G), aspartic acid (D), glutamic acid (E), and valine (V) consistently had AEI > 1 across most species, suggesting a higher prevalence of these amino acids in highly expressed proteins. Conversely, tryptophan (W), leucine (L), histidine (H), serine (S), and cysteine (C) showed the opposite trend; tryptophan exhibited the lowest average AEI among the 88 species (mean value 0.83). Moreover, tryptophan was identified as the amino acid with the highest synthesis cost (cost  =  75.5),^[^ ^18^ ^]^ suggesting a detrimental effect on the soluble expression of proteins.

Next, we defined SRAB (see Section 4.3 Amino acid and protein expression level connection), a mathematical model calculated from the AEI, to approximate a linear fit for each protein to its expression in a particular species. Spearman's correlation coefficients between the abundance of all proteins in the original PaxDB data set (without protein selection) and their SRAB values for these 88 species were calculated. The results revealed a significant correlation, with Spearman correlation coefficient values ranging from 0.2 to 0.6 (average value = 0.46), between protein abundance and SRAB value across the 88 species (Figure 3b; Figure S1, Supporting Information). Thus, for proteins with unknown expression levels in specific hosts, the SRAB value can be computed using the host organism's AEI to provide an initial qualitative assessment of its expression level; a higher SRAB value indicates a greater likelihood that the protein has higher soluble expression in the corresponding host organism.

### 2.4 Protein Pretraining Model

To develop an AI model capable of deeply learning the relationship between amino acid sequence and the soluble expression level of a protein, we introduced the pretraining model MP‐TRANS. It is based on a transfer learning strategy, which has been proven effective in using knowledge from large‐scale datasets to improve the performance of specific downstream tasks.^[^ ^19^ ^]^ In the case of limited labeled data for the target task, the model can benefit from the rich feature representations learned during pretraining on a broad and diverse dataset. First, we constructed a data set based on the UniRef50^[^ ^20^ ^]^ database provided on the UniProt FTP site as of June 26, 2023, which contains a total of 59 142 917 protein sequences; the shortest and longest sequences contain 11 and 45354 amino acid residues (AAs), respectively. All sequences were used to train the MP‐TRANS model. For each sequence, the model can receive up to 1022 AAs (the maximum input sequence length of the model is 1024 minus 2 special Tokens “[CLS]”, “[EOS]”). Within the UniRef50 pre‐training dataset, sequences longer than 1022 amino acids retain the first 1022 amino acids from the N‐terminus for model training, representing 2.6% of the dataset. The remaining sequences, shorter than 1022 amino acids and comprising 97.4% of the dataset, are padded to a length of 1022 using the special token “[PAD]” for subsequent training purposes (Figure S2, Supporting Information).

The pre‐training of MP‐TRANS involved utilizing 8 Ascend NPU cards. The MP‐TRANS network design incorporates 8 Transformer layers, each featuring a multi‐head attention mechanism with 16 heads, a hidden size of 1024, and a sequence length of 1024. The design decisions were made with the goal of maximizing model capacity while also ensuring that the model can be fine‐tuned on a single NPU card.^[^ ^21^ ^]^ A sequence length of 1024 allows most sequences to be fully input into the model. The total number of parameters is ≈87 164 000 (Table S1, Supporting Information). This parameter count is optimized to achieve the maximum model size suitable for single‐NPU fine‐tuning. The encoder and Transformer components of the pretrained output model serve as the initial weight parameters for fine‐tuning subsequent models.

### 2.5 Construction of the Protein Expression Level Prediction Model MPB‐EXP

**Figure 4:**
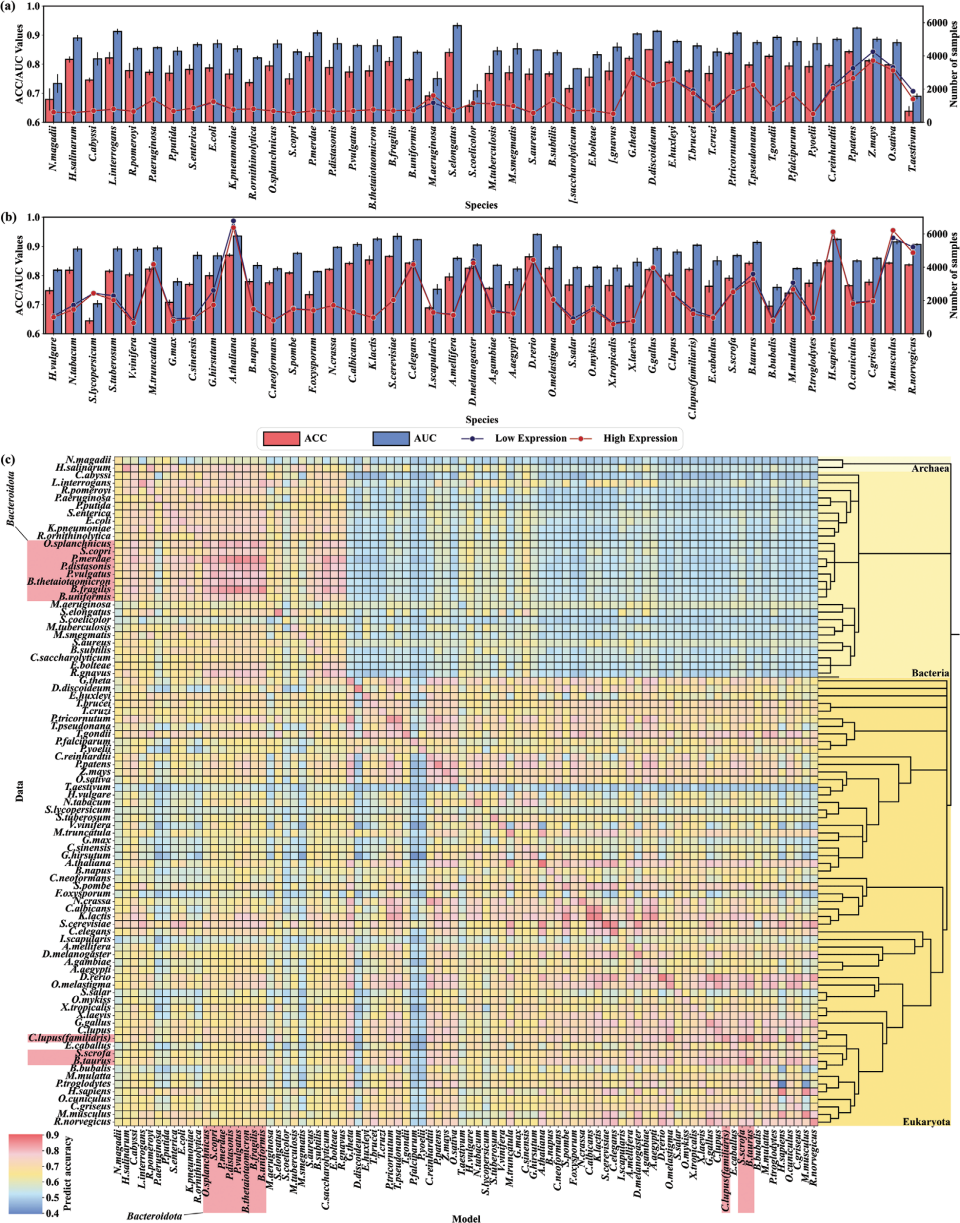
MPB‐EXP Model Evaluation and Cross‐Prediction Results. a, b) Accuracy (ACC) and area under the receiver operating characteristic curve (AUC) of the MPB‐EXP model for each of the 88 species on their respective test sets, error bars represent standard deviation. c) Cross‐prediction results across the 88 species.

For the previously selected core data set of 88 species, we fine‐tuned the MP‐TRANS pretrained model using a fivefold cross‐validation data set that corresponded to each species, yielding 88 fivefold cross‐validation models (termed MPB‐EXP models, as explained below). After the completion of model training with fivefold cross‐validation, we performed predictions using the same independent test set; the average of the 5 results was regarded as the final outcome. Evaluation results obtained using the MPB‐EXP model showed an average accuracy of ≈0.78 (range: 0.64–0.87). Notably, models for commonly used model organisms, such as *E. coli*, *S. cerevisiae*, *Arabidopsis thaliana*, and *Homo sapiens*, all achieved prediction accuracies above 0.75, meeting the predictive capability threshold for protein expression level (**Figure** **4**a,b; Table S2, Supporting Information). Moreover, we found a significant positive correlation between data number and accuracy (Spearman correlation coefficient *r* = 0.45, *p* < 0.005), indicating that for expression‐level models, an increased amount of data for model training can partially enhance the predictive ability of the model (Table S2, Supporting Information). Finally, we named the series of trained expression level models MPB‐EXP (MP‐TRANS‐based expression model).

Compared with other machine learning and deep learning methods, our MP‐TRANS approach achieved the best results in 3 evaluation species (*E. coli*, *Bacillus subtilis*, and *S. cerevisiae*), with an average accuracy ≈23.8% higher than conventional machine learning models, ≈8.6% higher than CNN, 5% higher than BiLSTM models, and 3% higher than the transfer learning method ESM+BiLSTM (**Table** **1**), these are commonly used protein prediction algorithms.^[^ ^22^ ^]^

**Table 1:** The comparison of the MPB‐EXP model with others on the datasets of *Escherichia coli*, *Bacillus subtilis* and *Saccharomyces cerevisiae*.

Species	Model	AUC	ACC	Recall	precision	F1	MCC
*E. coli*	MPB‐EXP	0.87 ± 0.014	0.79 ± 0.014	0.81 ± 0.031	0.78 ± 0.029	0.79 ± 0.01	0.58 ± 0.025
	SVM	0.57 ± 0.001	0.57 ± 0.001	0.61 ± 0.008	0.57 ± 0.001	0.59 ± 0.003	0.14 ± 0.002
	KNN	0.56 ± 0.001	0.56 ± 0.001	0.5 ± 0.002	0.58 ± 0.001	0.54 ± 0.002	0.12 ± 0.002
	GBDT	0.59 ± 0.002	0.59 ± 0.002	0.64 ± 0.0	0.59 ± 0.002	0.61 ± 0.001	0.17 ± 0.003
	CNN	0.79 ± 0.005	0.71 ± 0.01	0.76 ± 0.092	0.7 ± 0.038	0.73 ± 0.029	0.43 ± 0.015
	BiLSTM	0.82 ± 0.003	0.76 ± 0.004	0.83 ± 0.038	0.73 ± 0.014	0.78 ± 0.009	0.52 ± 0.012
	ESM+BiLSTM	0.84 ± 0.011	0.76 ± 0.013	0.74 ± 0.045	0.78 ± 0.03	0.76 ± 0.017	0.52 ± 0.026
	non‐pre‐trained	0.84 ± 0.003	0.76 ± 0.007	0.81 ± 0.044	0.74 ± 0.012	0.77 ± 0.014	0.52 ± 0.018
*B.subtilis*	MPB‐EXP	0.84 ± 0.01	0.77 ± 0.009	0.76 ± 0.042	0.78 ± 0.033	0.77 ± 0.01	0.53 ± 0.02
	SVM	0.53 ± 0.002	0.53 ± 0.002	0.51 ± 0.008	0.53 ± 0.002	0.52 ± 0.005	0.05 ± 0.004
	KNN	0.53 ± 0.0	0.53 ± 0.0	0.42 ± 0.0	0.55 ± 0.0	0.48 ± 0.0	0.07 ± 0.0
	GBDT	0.57 ± 0.001	0.57 ± 0.001	0.58 ± 0.002	0.58 ± 0.001	0.58 ± 0.001	0.15 ± 0.002
	CNN	0.74 ± 0.012	0.67 ± 0.013	0.65 ± 0.049	0.68 ± 0.018	0.66 ± 0.024	0.34 ± 0.024
	BiLSTM	0.78 ± 0.005	0.71 ± 0.009	0.69 ± 0.099	0.73 ± 0.057	0.71 ± 0.027	0.43 ± 0.02
	ESM+BiLSTM	0.8 ± 0.003	0.72 ± 0.007	0.7 ± 0.022	0.73 ± 0.017	0.71 ± 0.007	0.43 ± 0.015
	non‐pre‐trained	0.79 ± 0.002	0.72 ± 0.012	0.68 ± 0.076	0.75 ± 0.029	0.71 ± 0.032	0.44 ± 0.017
*S.cerevisiae*	MPB‐EXP	0.93 ± 0.011	0.87 ± 0.004	0.91 ± 0.009	0.85 ± 0.004	0.88 ± 0.004	0.73 ± 0.009
	SVM	0.62 ± 0.001	0.63 ± 0.001	0.68 ± 0.003	0.64 ± 0.001	0.66 ± 0.001	0.25 ± 0.001
	KNN	0.54 ± 0.001	0.52 ± 0.001	0.33 ± 0.001	0.59 ± 0.002	0.42 ± 0.001	0.08 ± 0.002
	GBDT	0.63 ± 0.001	0.64 ± 0.001	0.69 ± 0.0	0.65 ± 0.001	0.67 ± 0.0	0.27 ± 0.001
	CNN	0.88 ± 0.006	0.79 ± 0.009	0.82 ± 0.03	0.8 ± 0.013	0.81 ± 0.011	0.59 ± 0.018
	BiLSTM	0.88 ± 0.001	0.81 ± 0.006	0.84 ± 0.021	0.81 ± 0.005	0.82 ± 0.008	0.62 ± 0.012
	ESM+BiLSTM	0.93 ± 0.004	0.86 ± 0.008	0.9 ± 0.029	0.85 ± 0.02	0.88 ± 0.008	0.73 ± 0.016
	non‐pre‐trained	0.89 ± 0.006	0.82 ± 0.007	0.87 ± 0.03	0.81 ± 0.021	0.84 ± 0.006	0.64 ± 0.013

**Three machine learning models**: SVM (Support Vector Machine), KNN (K‐NearestNeighbor), and GBDT (GradientBoosting Decision Tree); **Two classic deep learning models**: CNN (Convolutional Neural Network) and BiLSTM (Bi‐directional Long Short‐Term Memory); **A transfer learning model**: ESM+BiLSTM (based on Evolutionary Scale Modeling (ESM2) pre‐trained encoding and introducing BiLSTM as a hidden layer transfer learning model); **non‐pre‐trained model**: a model that uses the same network architecture as MPB‐EXP but does not use pre‐trained weight parameters, denoted as non‐pre‐trained model. The data is the mean ± standard deviation.

Notably, the 2 methods that use transfer learning, ESM+BiLSTM, and MPB‐EXP, achieved the best results among all the models. Compared with CNN and BiLSTM models, the use of ESM and MP‐TRANS as pre‐training methods increases in average accuracy of 3.8% and 6.8%, respectively. Also, we used the same MP‐TRANS architecture and fine‐tuned the initial weights of the model directly as the non‐pre‐trained model. Our pre‐trained MPB‐EXP model showed a 4.3% increase in accuracy compared to the non‐pre‐trained model (Table 1). The significance of the difference in the 5‐fold cross‐validation data between these models was assessed using the Mann‐Whitney U test. The *p*‐Values from Table S3 (Supporting Information) confirmed significant differences, highlighting the importance of transfer learning in training the MPB‐EXP model.

### 2.6 Cross‐Prediction Among Species‐Specific Models

Cross‐species prediction was used to explore connections between different species. Each of the 88 MPB‐EXP models was required to predict the independent test set without any interference from homologous sequences of other species (see Section 4.5 MP‐TRANS fine‐tuning model for expression level prediction). The results indicated that the MPB‐EXP models excelled in predicting their own species; they also exhibited strong predictive abilities for cross‐species prediction within the same kingdom. Among prokaryotes, cross‐predictions within Bacteroidota showed higher accuracy. Similarly, satisfactory cross‐prediction accuracy was demonstrated in eukaryotes. However, the cross‐prediction effect of MPB‐EXP models between eukaryotic and prokaryotic microorganisms exhibited substantial variability. Models of eukaryotes generally performed poorly when predicting data for prokaryotes, but models of prokaryotes maintained a prediction level of ≈0.7 for eukaryotes (Figure 4c). Therefore, we assumed that closely related organisms tend to have higher accuracy in cross‐species predictions relative to those with distant phylogenetic relationships. Closely related species have higher degrees of identity in protein sequences and structures, leading to similarities in biological processes and mechanisms, which result in comparable expression patterns. This similarity makes it easier for models to accurately predict protein expression levels.^[^ ^16^, ^23^ ^]^

### 2.7 Predictive Performance and Interpretability of MPB‐EXP for Xylanase Pm10868

To assess the predictive accuracy of MPB‐EXP models, the xylanase Pm10868 (GH10 family) derived from the genome of rumen ciliates was chosen as the experimental test protein.^[^ ^24^ ^]^ The MPB‐EXP models predicted that Pm10868 would be expressed as a soluble protein in eukaryotic cells but not prokaryotic cells, such as *E. coli*. The predicted high expression probability value (HE‐Value) of model MPB‐EXP *S. cerevisiae* was 0.93 for wild‐type, whereas the HE‐Value of model MPB‐EXP *E. coli* was 0.26; these findings were consistent with experimental results showing almost no expression in *E. coli* (Figure 6a) and successful expression in soluble form in yeast cells.^[^ ^24^ ^]^

**Figure 5:**
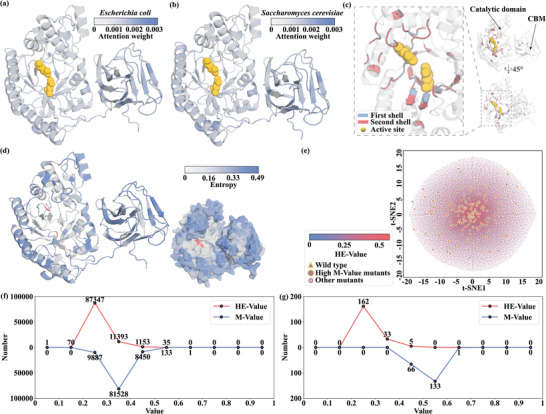
Analysis of generated mutants. a) Average attention distribution of the MPB‐EXP *E. coli* model for xylanase Pm10868. b) Average attention distribution of the MPB‐EXP *S. cerevisiae* model for xylanase Pm10868. c) Main structures of xylanase Pm10868, including the locations of CBM, active site, first shell, and second shell. d) Entropy values at various positions for the generated mutants. e) t‐SNE dimensionality reduction results of the generated mutant sequences. HE‐Values are represented by colors, wild‐type values are represented by triangles and mutant values are represented by dots or larger yellow circles (high M‐Value). Where the yellow circles represent the 200 high M‐Value mutants screened, most of these mutants are close to the wild‐type Pm10868 represented by the triangle symbol in the center. f) Distributions of HE‐Value and M‐Value for the 100000 generated mutants. g) Distributions of HE‐Value and M‐Value for the 200 selected high M‐Value mutants.

To enhance interpretability, gain further insights concerning the ability of the model to determine high or low expression, and determine which part(s) of the molecule may affect expression, we analyzed the regions of Pm10868 where MPB‐EXP *E. coli* and *S. cerevisiae* models were focused (**Figure** **5**a–c). As expected, the results demonstrated that the 2 host models primarily focused attention on the CBM domain in the N‐terminal region and regions distant from the active site (distal sites). Furthermore, the model assigned the lowest weight of attention to the active site, followed by the First shell and then the Second shell (conserved regions around the active site).^[^ ^25^ ^]^ The attention weight became lower with increasing proximity to the active site. In summary, the MPB‐EXP can concentrate on specific regions with the potential to enhance expression level while maintaining integrity regarding crucial functional areas.

### 2.8 Construction of Mutant Generation Model for Enhancing Pm10868 Expression in *E. coli*

To further validate the applicability and effectiveness of the MP‐TRANS and MPB‐EXP models, we introduced another mutant generation model, MPB‐MUT (mutation generation model based on MP‐TRANS); this model increased the heterologous expression levels of the target protein, using Pm10868 expressed in *E. coli* as an example. Initially, a search of the UniRef90 data set yielded 11645 homologous sequences for xylanase Pm10868, 86.9% of which were explicitly annotated as xylanases. Using these sequences, the mask‐based generation model, MPB‐MUT, generated 100000 mutations based on the wild‐type Pm10868.

To analyze the rationality of the 100000 generated sequences, the entropy values of all mutations were calculated to determine the frequency of occurrence of specific amino acids at specific positions. Model‐designed mutations were predominantly located on the protein surface; minimal alterations occurred in the internal TIM‐barrel on the main chain (Figure 5c,d; Figure S3, Supporting Information). Further analysis revealed that no mutations were located at the 2 active sites (314E and 413E) in Pm10868; additionally, the amino acids around these 2 sites in the protein structure were rarely mutated. Notably, mutations more frequently occurred at distal sites, such as the CBM domain and surface, with higher mutation entropy values. These findings were consistent with the high attention weights of the MPB‐EXP model, indicating that both the MPB‐MUT and MPB‐EXP AI models learned amino acid features that affect the soluble expression of the protein while preserving its function. For further analysis of the mutation directions of the above 100000 sequences, we used t‐SNE^[^ ^26^ ^]^ to reduce the dimensionality of these sequences and display their distribution in 2D space (Figure 5e). The wild type was in the very center of the entire t‐SNE, whereas mutants generated by MPB‐MUT uniformly extended in all directions, indicating that MPB‐MUT can search for mutants across the entire sequence space.

We then assessed the mutation confidence value (M‐Value) of the entire sequence using the MPB‐MUT model, along with the high‐expression probability (HE‐Value) predicted by the MPB‐EXP *E. coli* model for the mutants (Figure 5f). Most mutants exhibited M‐Values ranging from 0.3 to 0.4; only 133 mutants showed higher values within the range of 0.5–0.6 (Figure 5f). A higher M‐Value indicated a greater likelihood of retaining natural protein functionality, and mutants with high M‐Values were positioned closer to the wild type in sequence space (Figure 5e). The HE‐Values of most mutants were clustered ≈0.2–0.3 and centered around the wild‐type HE‐Value of 0.26, whereas 1188 mutants had elevated HE‐Values (0.4–0.6) (Figure 5f), indicating a greater likelihood of high expression compared with the wild type. We selected the top 200 mutant sequences based on M‐Value scores within the range of 0.49–0.61 (Figure 5g).

Subsequently, the MPB‐EXP *E. coli* model was used to predict the expression levels of these 200 mutant sequences, and 3 mutants with the highest HE‐Values were selected for experimental validation (**Table** **2**). The wild‐type Pm10868 protein sequence and the 3 mutant sequences are shown in Table S4 (Supporting Information). Compared with the wild type, the SRAB values of the 3 selected mutants were slightly increased, consistent with our methodology for protein expression profiling, based on sequence analysis.

**Table 2:** Selection indexes and SRAB values for xylanase wild type and mutants.

Protein	M‐Value	HE‐Value	SRAB
Pm10868	–	0.26	1.80
Pm10868‐85	0.50	0.49	1.80
Pm10868‐11	0.50	0.48	1.83
Pm10868‐04	0.50	0.47	1.82

M‐Value: Mutation Confidence Value; HE‐Value: High‐Expression Probability; SRAB: Strength of Relative Amino Acid Bias. All values are calculated using the Amino Acid Expression Index. The proteins included are the wild‐type Pm10868 and 3 model‐generated mutants.

### 2.9 Determination of Expression Level and Enzyme Activity of Xylanase Mutants

**Figure 6:**
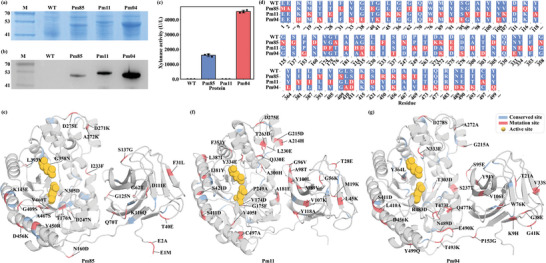
Experimental validation and analyses of the mechanisms underlying expression level enhancement in 3 Pm10808 mutants. a) Detection of soluble expression in the supernatant by SDS‐PAGE. b) Detection of soluble expression in the supernatant by Western blotting. c) Wild‐type (PM) and Pm10868‐11 (PM11) showed no enzyme activity, error bars represent standard deviation. d) Mutation sites in the sequence. e) Mutation sites in Pm10868‐85. f) Mutation sites in Pm10868‐11. g) Mutation sites in Pm10868‐04. In the figure, WT refers to the wild‐type Pm10868 protein, Pm85 refers to the mutant Pm10868‐85, Pm11 refers to the mutant Pm10868‐11, and Pm04 refers to the mutant Pm10868‐04.

The 3 selected mutants and wild‐type Pm10868 were heterologously expressed in *E. coli* BL21(DE3), the nucleic acid sequences optimized for *E. coli* codons are shown in Table S5 (Supporting Information); their expression levels were determined by SDS‐PAGE and Western blotting analysis. The results showed that the wild‐type Pm10868 was not expressed in *E. coli*; in contrast, the mutants Pm10868‐85, Pm10868‐11, and Pm10868‐04 were all expressed in soluble form in *E. coli*, and the levels of soluble expression gradually increased (**Figure** **6**a,b). In particular, the mutant Pm10868‐04 achieved almost complete soluble expression (Figure S4, Supporting Information). Pm10868‐04 showed the highest activity of 4554.59 ± 90.37 U/L, and Pm10868‐85 showed activity of 1609.93 ± 88.87 U/L. Although the soluble expression level of mutant Pm10868‐11 was increased, it showed no enzyme activity (Figure 6c). These results showed that the combination of mutations designed by the MPB‐MUT model with predictions from the MPB‐EXP model can effectively increase the soluble expression of xylanase Pm10868 in *E. coli*; all 3 expression‐enhancing mutants were consistent with the predictions of MPB‐EXP.

Compared with the wild type, Pm10868‐04 had 25 mutated sites, 19 of which were mutated toward amino acids with AEI > 1. Pm10868‐11 had 19/24 and Pm10868‐85 had 11/23 (Figure 6d and Figure 3a), confirming that amino acids with high AEI values can effectively improve soluble expression. K, D, and E are charged amino acids that increase hydrophilicity and contribute to correct protein folding.^[^ ^27^ ^]^ Due to their small side chain residues, A, G, and V can be easily embedded into the tertiary structure of proteins, minimizing steric hindrance and ultimately contributing to the correct folding of the target protein^[^ ^28^ ^]^ (Figure 6e–g).

### 2.10 Determination of Expression Level of Designed Cellulase and PETase

**Figure 7:**
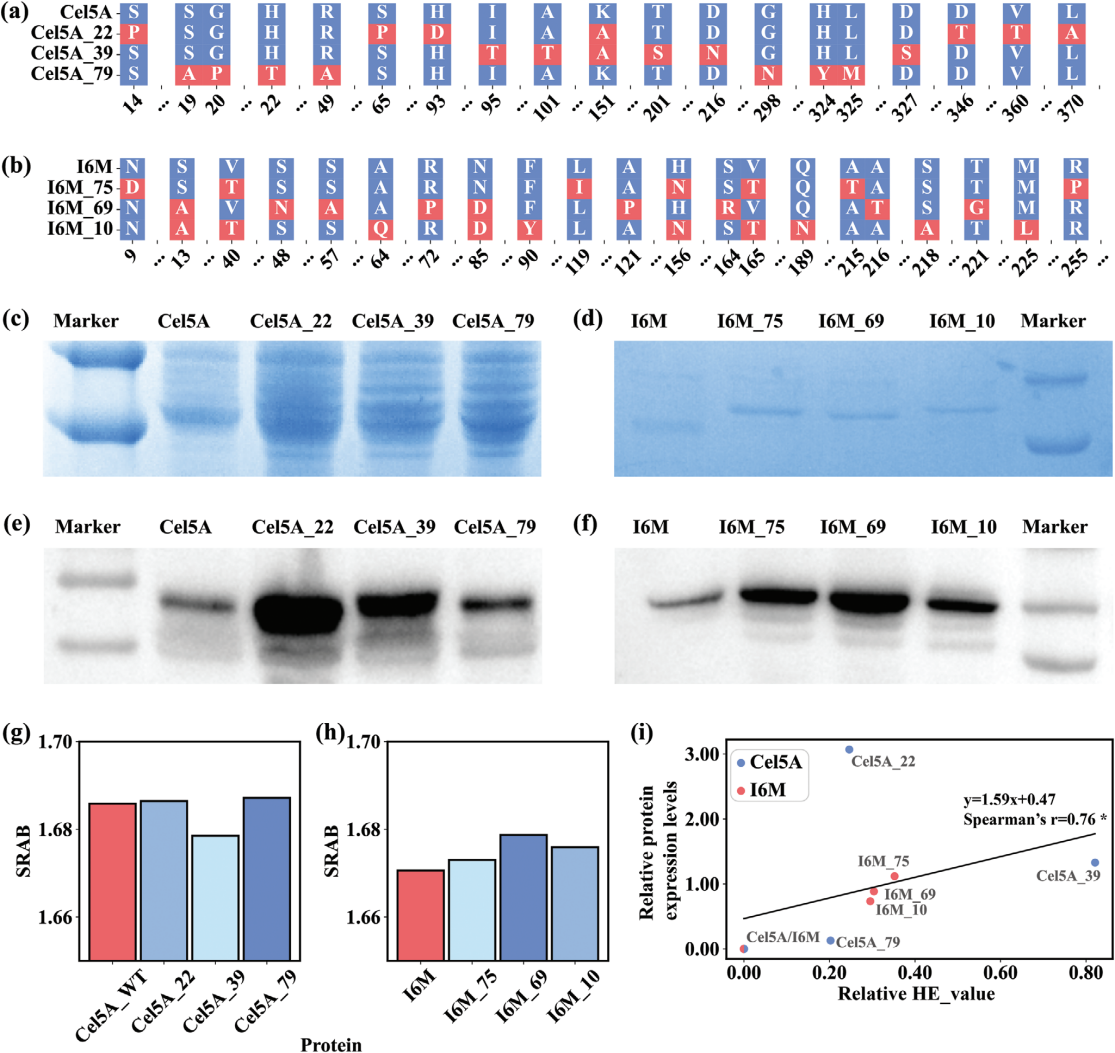
Experimental validation and analyses of the mechanisms underlying expression level enhancement in cellulase and PETase. a, b) Mutation sites in the sequence of cellulase Cel5A and PETase LCCICCG_I6M. c, d) Detection of soluble expression of cellulase Cel5A and PETase LCCICCG_I6M in the supernatant by SDS‐PAGE. e, f) Detection of soluble expression of Cel5A and PETase LCCICCG_I6M in the supernatant by Western blotting. g, h) The Sequence‐Richness Alignment Bias (SRAB) value was determined for mutants of cellulase Cel5A and PETase LCCICCG_I6M. i) Correlation between predicted high‐level expression propensity (x‐axis) in *E. coli* and actual protein expression levels (y‐axis) determined empirically through western blot analysis. The value in the plot represents the relative value, which is determined using the formula (x(mutant)‐x(wt))/x(wt).

To further test the widespread application of the pipeline, mutations were designed and screened for the cellulase Cel5A (NCBI Reference Sequence: XP_0 036 59014.1) and the PET plastic‐degrading enzyme LCCICCG_I6M,^[^ ^29^ ^]^ apart from xylanase Pm10868, using a consistent protocol to enhance soluble expression in *E. coli*. The top 3 predicted values for Cel5A mutants were 0.998 (Cel5A_39), 0.683 (Cel5A_22), and 0.659 (Cel5A_79) (Table S6, Supporting Information). Similarly, for the PET plastic‐degrading enzyme LCCICCG_I6M, the top 3 predicted values for mutants were 0.476 (I6M_75), 0.459 (I6M_69), and 0.456 (I6M_10) (Table S6, Supporting Information). The relevant protein sequences and their codon‐optimized gene sequences are shown in Tables S7–S10 (Supporting Information). The distribution of mutated sites of these mutants is shown in **Figure** **7**a,b. Evaluation of mutant expression levels in *E. coli* through SDS‐PAGE and Western blot analyses revealed varying degrees of enhancement, with notable improvements observed for Cel5A‐22 and I6M‐69 compared to their respective wild types, as illustrated in Figure 7c–f.

We also applied the Sequence‐Richness Alignment Bias (SRAB) metric to analyze mutants, finding that 5 out of 6 mutants showed higher values than their respective wild types (Figure 7g–h). Additionally, we examined the relationship between predicted high‐level expression propensity in *E. coli* and actual protein expression levels determined empirically through western blot analysis. The calculated Spearman's correlation coefficient was ≈0.76 (Figure 7i), *p *= 0.03, indicating a significant positive correlation. These results imply that our approach can be valuable in directing the design of mutant variants to improve protein expression levels.

## 3 Discussion

This study introduced a novel pipeline integrating AI models to predict and enhance the soluble expression levels of target proteins in heterologous hosts. Through data analyses, we found that soluble protein expression is closely related to amino acid biosynthesis cost, charge, and side chain. Therefore, we developed the expression prediction model MPB‐EXP for 88 species and the mutant generation model MPB‐MUT to further enhance soluble target protein expression. Both AI models effectively learned the amino acid features that affect protein soluble expression while preserving functionality across different species. This comprehensive solution focuses on hosts and amino acid sequences; it more effectively addresses the challenges of predicting and improving soluble expression compared with conventional protein engineering methods and existing machine learning methods, such as CNN, LSTM, and ESM+LSTM models based on transfer learning.

According to Alon Diament's work, the 3D eukaryotic genomic organization is strongly correlated with codon usage, expression, and function, but not monotonically related to the amino acid frequencies in *Saccharomyces cerevisiae*, *Schizosaccharomyces pombe*, *Arabidopsis thaliana*, mouse and human.^[^ ^30^ ^]^ In this study, our developed pre‐trained model effectively captured protein sequence features beyond amino acid frequencies, enabling the training of a protein expression prediction model based on this constructed pre‐trained model. Through an analysis of protein abundance and sequences from the PaxDB5.0 dataset, we computed 20 amino acid enrichment index (AEI) values for each of the 88 species. Higher AEI values were found to correlate with increased soluble expression levels. These AEI values were then utilized to predict and quantify the relationship between protein expression and sequence using the Sequence‐Richness Alignment Bias (SRAB) metric. Notably, the SRAB values of the 3 selected mutants exhibited a slight increase compared to the wild type. Collectively, both the calculated and experimental outcomes underscored the encoding of protein expression information within the protein sequence, thereby suggesting its potential utility in designing protein expression in the host system.

In addition, we use the protein sequences sourced from the previous work conducted by Vogel et al.^[^ ^31^ ^]^ and Zur et al.^[^ ^32^ ^]^ to train the linear regression MPB‐EXP‐R model. The results demonstrated that the simulated correlation coefficients between the predicted protein abundance and experimental data were ≈0.65 for both datasets (Figure S5, Supporting Information), slightly lower than the correlation coefficients of 0.67 and 0.70 reported in the respective studies. It is worth noting that Christine Vogel's work,^[^ ^31^ ^]^ incorporated the codon usage, 3 ′UTR and 5′ UTR features, and secondary structures, while Hadas Zur's study^[^ ^32^ ^]^ utilized codon usage, Predicted Folding Energy, ATG Context Score, as well as 5 ′UTR features to train their models. Despite the utilization of solely protein sequence data, our approach still achieved a robust positive correlation coefficient, indicating that our model is capable of effectively capturing protein expression information from protein sequences.

Furthermore, we developed a regression model, MPB‐EXP‐R, following the work of Vogel et al.^[^ ^31^ ^]^ and Zur et al.,^[^ ^32^ ^]^ using a log‐scale processed PaxDB protein abundance dataset. This model was fine‐tuned on the pre‐trained model MP‐TRANS for 88 species. Our prediction performance consistently demonstrated a strong correlation, with correlation coefficients ranging from 0.28 to 0.8 and an average coefficient of 0.64 (Additional result 1). These results further support the notion that protein sequences contain valuable information regarding protein expression levels.

Consistent with Filip Buric's work,^[^ ^33^ ^]^ altering the sequence of amino acids can potentially influence protein expression and enzyme activity, with the outcome largely determined by the specific location and orientation of the mutation. Our model maximizes the avoidance of affecting enzyme activity through the self‐attention mechanism. Take xylanase Pm10868 as an example, the distribution of attention in MPB‐EXP and the mutation sites in MPB‐MUT showed that despite the diversity of the 100000 sequences generated by MPB‐MUT, the 2 models unanimously focused on the CBM region and areas distant from the active site of Pm10868. Both models aimed to maximize the prediction and improvement of soluble expression while maintaining maximal functionality. This was likely because amino acid residues in the active region are highly conserved for the structure and catalytic activity of the protein. The models likely learned that variations in these regions have minimal impact on expression level and therefore did not require excessive attention. Among the 3 mutants we screened, the expression levels were all increased, and in terms of enzyme activity, Pm10868‐04 had the highest enzyme activity, followed by Pm10868‐85, and Pm10868‐11 had no enzyme activity. The analysis of the mutation sites of Pm10868‐11 showed that compared with the other 2 mutants, these mutation sites were more densely distributed around the active site, which may have resulted in loss of activity. However, as shown in Table S11 (Supporting Information), Pm10868‐11 received the lowest attention at the active site, first shell, and second shell, whereas it received excessively high attention in the CBM domain. This may have been because the model has not yet achieved a balance between soluble expression of the protein and functional preservation, indicating a direction for further improvement of model accuracy.

Finally, although this article focused on xylanase as an example to analyze the detailed mechanisms of the model, the analysis revealed that our methods were also effective with PETase and cellulase. The expression of soluble proteins in different hosts is influenced by several factors. In future research, we aim to incorporate additional biological characteristics, including codon usage and secondary structures, into our model training to improve its predictive and modification capacities. Furthermore, we will use the same transfer learning approach to integrate predictions of protein function or catalytic efficiency to ensure the functionality of the produced proteins.

## 4 Experimental Section

### Protein Expression Level Data Set Collection and Processing

The PaxDb 5.0 (Protein Abundances Across Organisms, https://pax‐db.org) database serves as a comprehensive meta‐resource of protein abundance levels in tissue‐specific or whole‐organism proteomes.^[^ ^17^ ^]^ Here, the whole protein abundance information from the PaxDB5.0 data set was downloaded. For the dataset of each species, the abundance of each sequence was ranked from high to low and evenly divided the entire dataset into 3 categories, designating them as relatively high, medium, and low abundance. Concurrently, the CD‐HIT^[^ ^34^ ^]^ tool was used to cluster protein sequences with a similarity of 90%. Next, the homologous sequences were discarded and the representative sequences of each cluster, which were only from the high or low abundance categories, were selected as described in Method S1 (Supporting Information). These sequences were then used to construct a relatively balanced dataset. Afterward, in order to ensure sufficient data for model training, species with more than 1000 data entries for analysis were only selected.

### Expression Level Data Analysis

For the protein expression level data set constructed as described above, factors affecting protein expression were analyzed levels from the perspective of the amino acid sequence through 2 analytical strategies: amino acid biosynthetic cost and amino acid index analysis.

The amino acid biosynthetic cost, derived from the work of Heizer et al.,^[^ ^18^ ^]^ is a fixed value corresponding to each amino acid based on the number of high‐energy phosphate bonds (∼P) involved in biosynthesis within *E. coli*. Additionally, due to the universality of amino acid biosynthetic pathways,^[^ ^18^, ^35^ ^]^ these costs are applicable to numerous organisms. The amino acid biosynthetic cost values as shown in Table S12 (Supporting Information). The average amino acid biosynthetic cost for each protein in the data set as the protein's synthesis cost was calculated.

The amino acid index was derived from the Amino Acid Index (AAindex) database,^[^ ^36^ ^]^ which focuses on numerical indices of various physicochemical and biochemical properties of amino acids and amino acid pairs (i.e., representing the 20 amino acids with 20 numerical values based on a specific property). Here, “AAindex1” was used from AAindex database version 9.2, which is an index of 20 numerical values for amino acids, including those for 566 properties. This is the core part of the AAindex database, representing a specific physicochemical or biological property such as hydrophobicity, hydrophilicity, polarity, volume, and conformation parameters. In each entry of AAindex1, detailed information and annotations are provided, including the unique identification number of the entry, a brief description, the PubMed identification number, etc., as well as the actual index data used in this study for calculation, listing the values of the 20 amino acids in a fixed order.

For analysis of amino acid index data, to facilitate comparisons among amino acid properties, Z‐score normalization on the selected properties relative to index values of the 20 amino acids was performed. This standardized each property according to the following formula:

(1) $$Z=\frac{\left(X-\mu \right)}{\sigma }$$

where X is the original index value, μ is the mean of the index values for the property, and σ is the standard deviation of the index values for the property. Through normalization, these properties could be compared within the same range. The comparison method used hierarchical clustering applied to the selected key properties (horizontally) and the 20 amino acids (vertically). By calculating the similarity between each property or amino acid, groups were gradually merged or split to form a clustering tree.

Here, the approach to identify the index most strongly correlated with expression levels involved initially calculating the average index value for each sequence with respect to a given index, representing the index value for that protein. Subsequently, with respect to all proteins of a species in the protein expression level data set, protein index values using this method for the given index were calculated, and then Spearman's correlation coefficient between these index values and their expression levels. Finally, the correlation coefficients for this index across all species and identified as the index most strongly correlated with expression levels based on the average correlation coefficient were averaged.

The phylogenetic tree used in this study was generated with phyloT^[^ ^37^ ^]^ using NCBI Taxonomy^[^ ^38^ ^]^ and visualized with iTOL.^[^ ^39^ ^]^

### Amino Acid and Protein Expression Level Connection

Here, the AEI was defined to establish a quantifiable link between amino acid selection and expression levels. The AEI was calculated using the following formula for each species:

(2) $$\mathrm{AEI}\left(a\right)=\frac{\exp\left(\mathrm{freq}\left(a_{h}\right)\right)}{\exp\left(\mathrm{freq}\left(a_{l}\right)\right)}$$

The expression level data set to calculate AEI values was used. First, The frequency (freq) of each amino acid for every sequence was calculated. Then, for each amino acid, the expected amino acid frequencies were calculated in high‐expression proteins (a_h_) and low‐expression proteins (a_l_). Finally, the expected value for high‐expression proteins by the expected value for low‐expression proteins to determine the AEI for each of the 20 amino acids corresponding to each species was divided.

Subsequently, the SRAB was defined for individual protein sequences using the following formula to approximate a linear fit of the protein expression level:

(3) $$\mathrm{SRAB}=\exp\left(\frac{\sum_{i=1}^{n}\log\left(f\left(a_{i}\right)+1\right)}{n}\right)-1$$

The definition of the SRAB value was similar to measuring the relative codon bias strength (RCBS) of each protein abundance^[^ ^40^ ^]^ based on codon selection by calculating the geometric mean of AEI values of the protein in the target host organism; it could be used to qualitatively approximate the protein expression level in the target host. A higher SRAB value indicated that the amino acid usage in the protein sequence was more closely aligned with amino acids present at high abundance in the target host organism, suggesting that the protein had a relatively high expression level in the target host. Conversely, a lower SRAB value suggested a tendency toward a lower expression level.

### MP‐TRANS Model

The MP‐TRANS model architecture used here was built on the architecture of the Transformer,^[^ ^41^ ^]^ stacking multiple identical Transformer encoder layers in series and functioning as an autoencoder model. In this model, the output for each residue contained information from the entire sequence, reflecting the relative importance of all elements in the sequence with regard to the current element. This approach enabled MP‐TRANS to effectively capture long‐distance interactions and dependencies between residues in protein sequences, understand complex protein features, and increase model sensitivity to different types of potential features within proteins. Additionally, due to the parallelism of the self‐attention mechanism, MP‐TRANS showed higher computational efficiency compared with conventional recurrent neural network models.

The input layer of MP‐TRANS combined Token Embedding and Position Embedding to adapt to the analysis of single protein sequences. Token Embedding numerically represents amino acids and special tokens, capturing semantic features; because the Transformer architecture does not naturally process sequence order, Position Embedding provides supplemental sequence position information.

Pretraining primarily utilized the MLM (Masked Language Model) task (i.e., masking some amino acids in the sequence and predicting them), with a focus on model training to understand the context of protein sequences. Additionally, the output layer is a linear fully connected layer that converts the Transformer output into a probability distribution with a size equal to the vocabulary, processed by the Softmax function to predict masked tokens, using cross‐entropy loss and the AdamWeightDecay optimizer for training. The specific construction method for the pretraining task is described in Method S2 (Supporting Information).

The pretraining data set for MP‐TRANS was sourced from the UniRef50 database^[^ ^20^ ^]^ on the UniProt website. MP‐TRANS utilized self‐supervised pretraining; during the construction of the pretraining set, protein sequences were treated as sentences, amino acids were treated as tokens, and tokens were randomly masked with 15% probability. The masking method included an 80% chance of using the special token “[MASK],” a 10% chance of using a random amino acid, and a 10% chance of leaving the amino acid unchanged. Special tokens “[CLS]” and “[EOS]” were added at the beginning and end of sequences, respectively, with the maximum sequence length set to 1024 tokens (containing a maximum of 1022 AAs). Sequences not reaching this length were padded with “[PAD],” sequences exceeding this length were truncated, and nonstandard amino acids were replaced with “[UNK].” The constructed “vocabulary” included 20 types of amino acids and 5 special tokens. The specific method for constructing the pretraining data set is shown in Method S3 (Supporting Information).

MP‐TRANS was written in Python and runs on the Huawei MindSpore version 1.8 deep learning framework, which is based on Python 3.8. The training environment operates on Docker. MindSpore is an open‐source deep learning framework that relies on the CANN 5.2 deep learning computing library, enhancing computing performance through built‐in efficient operators and optimization strategies. The hardware for training was the Huawei Atlas 800–9000 deep learning server, equipped with 8 Huawei Ascend 910B deep learning cards (Neural Processing Units, NPUs) to accelerate neural network computations; each card had 32 GB of memory. Additionally, the server included 4 Kunpeng 920 CPUs, with server memory totaling 24 × 32 GB. The server utilized the Galaxy Kirin server operating system V10, which is based on Linux kernel version 4.19.

### MP‐TRANS Fine‐Tuning Model for Expression Level Prediction

For MP‐TRANS model fine‐tuning, only minor modifications to the pretrained network were needed to construct a downstream fine‐tuning network that enabled protein expression level classification. Here, the input and hidden layers from the MP‐TRANS pretraining phase were retained; for the final output layer, from a fully connected network corresponding to each token to a network corresponding to the “[CLS]” token was switched, with an output dimension of 2, using the Softmax activation function. This setup was employed to classify high‐expression proteins (labeled as 1) and low‐expression proteins (labeled as 0).

The training data were the previously constructed core data set for protein expression levels: 20% of the data were set aside as an independent test set, and the remaining data were divided for fivefold cross‐validation. During the training process,5 models were separately trained on the fivefold cross‐validated data. To evaluate the classification model, the following metrics were used: accuracy (ACC), precision, recall, F1 score, Matthew's correlation coefficient (MCC), and area under the receiver operating characteristic curve (AUC).

Here, 3 machine learning models were constructed—Support Vector Machine (SVM), K‐Nearest Neighbors (KNN), and Gradient‐Boosting Decision Tree (GBDT)^[^ ^42^ ^]^—and 2 standalone deep learning models—Convolutional Neural Networks (CNN) and Long Short‐Term Memory (LSTM). A feature‐based transfer learning model was also constructed, ESM‐LSTM, which encoded sequences using the Meta ESM2 (Evolutionary Scale Modeling) protein encoder and generated classification results through an LSTM network.^[^ ^22^, ^43^ ^]^ These 6 methods in a data set using *E. coli*, *B. subtilis*, and *S. cerevisiae* with MPB‐EXP were systematically compared and evaluated. In evaluating MP‐TRANS and all its comparison models, the consistency of the dataset was strictly ensured by using pre‐divided and identical training, validation, and independent test sets to ensure that there is no bias in the comparison due to the data. The Grid Search method provided by the scikit‐learn package for traditional machine learning methods to find the optimal parameters was used. For deep learning methods, the Hyperas tool^[^ ^44^ ^]^ to select the optimal parameter combination through 1000 iterations was used.

In addition, to assess the cross‐species predictive ability of the model and explore the relationships between protein expression levels in different species, a cross‐prediction analysis was conducted using MPB‐EXP models. Specifically, for each MPB‐EXP model of all 88 species, we tasked it with predicting the data in the independent test set of all remaining species. Here, in order to avoid false positives caused by homologous sequences in different species, the CD‐HIT tool was used to cluster the dataset of each predictive model and the independent test set data of the remaining models. Finally, those sequences in the independent test set that were not clustered in the predictive model were selected. The method to 88 species and computed the cross‐prediction by clustering 7569 times was conducted.

As a supplement, a linear regression MPB‐EXP‐R model was also constructed. MP‐TRANS as the pre‐trained model, MSE as the loss function, and RMSE, R2, and Spearman's correlation coefficient as the evaluation criteria for the model were again used.

### Attention Weight Analysis of MPB‐EXP

The reasons underlying the model's differentiation of protein expression levels by extracting attention from the MPB‐EXP model were analyzed, specifically identifying which parts the model considered more relevant to protein expression. Here, the attention shape for the MPB‐EXP was Fold (5) × Layer (8) × Head (16) × Query (1024) × Key (1024), and the attention extraction strategy for each dimension was as follows:

*Fold*: Average the attention extracted from 5 cross‐validation models

*Layer*: Select the last layer (i.e., the eighth layer of the Transformer)

*Head*: Average the attention weights output by Softmax across 16 attention heads

Using the strategies outlined above, a 1024 × 1024 matrix corresponding to Query and Key was obtained. Then, the first row of attention (i.e., how the “[CLS]” token was extracted, used to integrate classification information, allocated its attention to other parts of the sequence [Query line of CLS]); thus, sequence information for classification decisions was summarized.

The structures shown for Pm10868 and its mutants were predicted by ColabFold^[^ ^45^ ^]^ based on Alphafold2.^[^ ^46^ ^]^ These PDB files were visualized and displayed using PyMOL.^[^ ^47^ ^]^

### MP‐TRANS Fine‐Tuned Model for Mutant Generation

The fine‐tuning method for the MP‐TRANS mutant design model was also based on retaining the input and hidden layers while defining a new output layer as the new network architecture. Here, the MLM method was used to achieve mutations at certain sites on the sequence.

For training data acquisition, the UniRef90 database was selected as the sequence alignment library, using the Jackhammer tool from the HMMER local toolkit^[^ ^48^ ^]^ based on the hidden Markov model. HMMER has achieved excellent results in various studies, including those by Thomas A. Hopf et al.,^[^ ^49^ ^]^ for homology sequence searches. In the work, similar methodologies and parameters were utilized as described in their research to perform homology sequence searches. The wild‐type sequence was used as the query sequence; a match threshold set at 0.5 times the sequence length was used for the sequence search. This match threshold setting controlled the minimum score threshold for reporting global alignments, the minimum score threshold for reporting local alignments, the minimum score threshold for the next round of iterative searches, and the minimum score threshold for local alignments included within the next round of iterative searches; the set match threshold was regarded as the input for these 4 parameters.

The HMMER alignment result sequences served as training data, with a 10% random probability of masking AAs using the special token “[MASK]”; this allowed the model to focus on predicting AAs at the current masked position that were more biologically meaningful. The model trained with this constructed data set was the mutant design model uniquely corresponding to the current wild‐type sequence. Here, a new evaluation metric, “mutation rate,” is introduced, defined as 1 minus the ratio of model predictions back to the original amino acids at all masked positions, reflecting the proportion of mutations that the model can induce in masked parts of the current sequence.

### Mutant Design Process for Enhancing Protein Expression

After the trained MPB‐MUT model had been acquired, the wild‐type Pm10868 sequence was randomly masked by 10% of the AAs, and amino acids that had been masked were predicted; this operation was performed 100000 times. This number was chosen to maximize the exploration of the mutation space within the constraints of limited time and computational resources. Then, mutations occurred when the AAs predicted by the model did not match the corresponding AAs at the same positions in the wild‐type sequence. These 100 000 multipoint masks were reduced by the model to effectively cover the entire sequence space of mutations.

After the mutations had been completed, the entropy was used to effectively represent the diversity of amino acids at each specific position across all generated mutants. Entropy is a well‐established measure in information theory that captures the uncertainty or variability within a dataset. The mutation entropy values were calculated and tabulated using the following entropy calculation formula:

(4) $$S=-\sum_{i}\left(p_{i}\times \log\left(p_{i}\right)\right)$$

where *S* is the entropy value, representing the diversity or uncertainty of a certain residue position across the entire protein; *p*_i_ is the probability of the *i* type of amino acid occurring at the current position, obtained through the division of the occurrence count of a certain amino acid at that position by the total number of amino acid occurrences at that position. High entropy at a specific position indicates a diverse distribution of amino acids, suggesting that this position is tolerant to mutations. Conversely, low entropy signifies that the position is conserved, with certain amino acids being predominant. So, by evaluating entropy across all positions, regions were identified within the protein that are more amenable to variation. Including entropy as a metric provides a quantitative basis for assessing the mutation landscape, ensuring that the approach systematically explores viable mutant sequences.

To perform quality control on the generated sequences, selection was performed according to the probability values of AAs predicted by the model. Specifically, for each sequence, the probability values of the predicted AAs were sorted at all predicted sites, then used the minimum probability values of these AAs to represent the M‐Value for the entire sequence. Then, the representative probability values of 100000 sequences were sorted, and selected the top 200 sequences with the highest representative probability values as candidate multipoint mutants.

For these 200 mutants, the MPB‐EXP *E. coli* model prediction was used, then sorted according to output probability of high expression (HE‐Value). Next, the mutant with the highest HE‐Value as the target expression boosting mutant was chosen.

### Experimental Validation and Computational Analysis of Pm10868 Mutants

To verify the expression of the wild type and 3 mutants in the heterologous host *E. coli*, the expression of candidate mutants in *E. coli* was determined, as described in Method S4 (Supporting Information), and their enzyme activity through biological experiments, as described in Method S5 (Supporting Information).

### Statistical Analysis

All data underwent rigorous pre‐processing to guarantee their quality and compatibility for statistical analysis. Protein sequences were clustered using CD‐HIT^[^ ^34^ ^]^ with a 90% sequence identity threshold to remove redundant sequences and reduce homology bias. For amino acid index analysis, the selected properties were standardized using Z‐score normalization to allow for direct comparison between different amino acid properties.

The model's performance was validated using five‐fold cross‐validation and evaluated with metrics including ACC, precision, recall, F1 score, MCC, and AUC. Error bars in figures represent the standard deviation of the mean, presented as mean ± standard deviation (SD). In comparative Model Analysis, the Mann‐Whitney U test was employed to compare the performance metrics between different models in the fivefold cross‐validation data. A two‐sided test was used with an alpha level of 0.05.

Spearman's rank correlation coefficient was used to assess the monotonic relationships between protein abundance and factors such as average biosynthetic costs and amino acid index properties. Correlation coefficients (*r*) and associated 2‐sided *p*‐values were calculated, with *p *< 0.05 considered statistically significant. Levels of significance are indicated with asterisks: *p < 0.05, **p < 0.01, ***p < 0.001.

In experimental validation, enzyme activity assays and expression level determinations were performed in triplicate for each mutant and the wild type. The enzyme activity data were statistically analyzed using GraphPad Prism (version 9.0).

Statistical analyses and data processing were performed using the following software and tools. Python (version 3.8) was used for general data processing and analysis, while MindSpore‐Ascend (version 1.8) served as the deep learning framework. NumPy (version 1.21) was employed for numerical computations, and SciPy (version 1.7) was used for statistical tests such as Spearman's correlation and the Mann‐Whitney U test. For machine learning evaluations and model performance metrics, scikit‐learn was utilized (version 0.24). Data manipulation and analysis were conducted using Pandas (version 1.0), and data visualization, plotting, as well as the generation of heat maps and dendrograms were performed using Matplotlib (version 3.2) and Seaborn (version 0.11.1). Additionally, CD‐HIT (version 4.8.1) was used for clustering protein sequences, PyMOL^[^ ^47^ ^]^ (version 2.4.1) for visualizing protein structures, and HMMER^[^ ^48^ ^]^ (version 3.3.2) for homology sequence searches. Lastly, ColabFold^[^ ^45^ ^]^ for protein structure prediction was accessed.

## Conflict of Interest

The authors declare no conflict of interest.

## Author Contributions

T.L. and Y.Z. contributed equally to this work. J.T., H.Q.H., F.F.G., and B.L. conceived and coordinated the study and edited the paper; T.Y.L. and Y.Y.Z., performed the model construction, experimental validation, and original paper writing; Y.J.L., G.S.X., H.G., and P.T.W. participated in performing experiments and discussing the results; T.T., H.Y.L., N.F.W., and B.Y. supervised the project. All authors discussed the results and have given approval to the final version of the paper.

## Supporting information



Supporting Information

Supporting Information

Supporting Information

## Data Availability

All datasets, models and codes used in this study are available on GitHub: https://github.com/Thurs‐Lw/MPB‐EXP‐MUT.
