# Supplementary material for: Effective Gene Expression Prediction and Optimization from Protein Sequences
Source: Adv Sci (Weinh). 2025 Jan 9;12(8):2407664. doi: 10.1002/advs.202407664 (PMC11848636; doi:10.1002/advs.202407664)
Supplement: Supplementary file 1 — Supporting Information [file ADVS-12-2407664-s001.docx]

Supplementary Material

EFFECTIVE GENE EXPRESSION PREDICTION AND OPTIMIZATION FROM PROTEIN SEQUENCES

Tuoyu Liu^b,c,1^, Yiyang Zhang^b,1^, Yanjun Li^b^, Guoshun Xu^a^, Han Gao^a^, Pengtao Wang^b^, Tao Tu^a^, Huiying Luo^a^, Ningfeng Wu^b^, Bin Yao^a^, Bo Liu^b, *^, Feifei Guan^b, *^, Huoqing Huang^a, *^, Jian Tian^a, *^

a. State Key Laboratory of Animal Nutrition and Feeding, Institute of Animal Sciences, Chinese Academy of Agricultural Sciences, Beijing 100193, China

b. National Key Laboratory of Agricultural Microbiology, Biotechnology Research Institute, Chinese Academy of Agricultural Sciences, Beijing 100081, China

c. School of Life Sciences, Tianjin University, Tianjin 300110, China

^1^ These authors contributed equally to this work; ^*^ Corresponding authors

Contents

[Additional results 4](#_Toc184834072)

[Additional result 1: MPB-EXP-R model used to predict protein abundance values 4](#_Toc184834073)

[Supplementary Methods 5](#_Toc184834074)

[Supplementary Method 1: Process for constructing expression level dataset from PaxDB 5](#_Toc184834075)

[Supplementary Method 2: Pre-training task 8](#_Toc184834076)

[Supplementary Method 3: Pre-training Dataset and Data Processing 10](#_Toc184834077)

[Supplementary Method 4: Prokaryotic Expression and Crude Enzyme Preparation of PM10868 and Its Mutants 12](#_Toc184834078)

[Supplementary Method 5: Enzyme Activity Assay of Xylanase 13](#_Toc184834079)

[Supplementary Method 6: Enzyme Activity Assay of Xylanase 14](#_Toc184834080)

[Supplementary Figures 15](#_Toc184834081)

[Figure S1 15](#_Toc184834082)

[Figure S2 16](#_Toc184834083)

[Figure S3 17](#_Toc184834084)

[Figure S4 19](#_Toc184834085)

[Figure S5 20](#_Toc184834086)

[Figure S6 21](#_Toc184834087)

[Figure S7 22](#_Toc184834088)

[Supplementary Tables 23](#_Toc184834089)

[Table S1 23](#_Toc184834090)

[Table S2 24](#_Toc184834091)

[Table S3 32](#_Toc184834092)

[Table S4 33](#_Toc184834093)

[Table S5 35](#_Toc184834094)

[Table S6 39](#_Toc184834095)

[Table S7 40](#_Toc184834096)

[Table S8 42](#_Toc184834097)

[Table S9 46](#_Toc184834098)

[Table S10 47](#_Toc184834099)

[Table S11 50](#_Toc184834100)

[Table S12 51](#_Toc184834101)

[Table S13 52](#_Toc184834102)

[Table S14 56](#_Toc184834103)

[Supplementary References 57](#_Toc184834104)

# Additional results

## Additional result 1: MPB-EXP-R model used to predict protein abundance values

For MPB-EXP, in addition to the classification model used in the main text, we additionally trained a linear regression model MPB-EXP-R. We still included the 88 species covered in the main text. For the data from PaxDB for each species, we first used the CD-HIT tool[1] to remove homologous sequences with 90% sequence similarity, and deleted entries with a protein abundance of 0. In addition, we used a log-scale method similar to Vogel's[2] work to log 10-transform the protein abundance of each sequence to obtain labels for training the model.

We again set aside 20% of the data as an independent test set and used the remaining 80% to train a five-fold cross-validation model. During model training, we again used MP-TRANS as the pre-trained model, MSE as the loss function, and RMSE, R2 and Spearman's correlation coefficient as the evaluation criteria for the model. The evaluation results of the 88 models contained in MPB-EXP-R on the independent test set are shown in Figure S6 and Table S13. The average RMSE of the models was 0.75, the average R2 was 0.41, the average Pearson correlation coefficient was 0.64, and the average Spearman correlation coefficient was 0.63. The models can roughly predict the expression levels of proteins at the overall level and can be used as a supplement to MPB-EXP.

# Supplementary Methods

## Supplementary Method 1: Process for constructing expression level dataset from PaxDB

In the PaxDB database, abundance values are calculated by processing mass spectrometry data. The database recalculates protein abundances using a method based on spectral counts, converting the frequency of protein detection into relative abundance values to provide an estimate of the protein's presence in the sample. Additionally, PaxDB performs data quality control through the STRING database[3]. Since interacting proteins tend to be expressed in roughly similar abundance ranges, the PaxDB database uses global protein-protein interaction information to estimate the data quality of each dataset included for each species, assigning an "interaction Z-score" as the evaluation score for each dataset. We downloaded the PaxDB5.0 protein abundance dataset "All datasets," its corresponding "Protein sequences," and the "UniProt mappings" file of STRING ID to UniProt ID from PaxDB's data download site (https://pax-db.org/download).

Our process for handling PaxDB data is shown in Figure 1(a). The PaxDB data downloaded contains multiple species; for each species, the first step is dataset selection. If a species contains only one dataset (for example, Anopheles gambiae contains only one dataset named 7165-GPM_201408), that dataset is chosen as the representative dataset for that species. However, when a species contains many different datasets (for example, Arabidopsis thaliana contains 59 datasets), the primary principle for selecting a representative dataset is coverage, not the highest scoring dataset. The rule is: WHOLE-integrated dataset > any tissue-integrated dataset (*-integrated) > the highest coverage dataset of all individual datasets. For example, if there are two datasets for Flower, but only one for Seed, since there are two Flower datasets, PaxDB will have an integrated dataset for Flower, and the Seed dataset does not have an integrated dataset. Then, the "Flower-integrated" dataset would be chosen, i.e., the integrated data of the two Flower datasets, even if the Seed dataset has a higher quality score. This method of dataset selection is the same as the filtering method displayed on the PaxDB website.

In these datasets, we represent the expression level of each protein with its protein abundance value. Next, for the selected datasets of each species, we sort the protein abundance values from highest to lowest. Based on the sorting results, the top 1/3 abundance proteins are marked as high-expression level proteins, the middle 1/3 as medium-expression level proteins, and the bottom 1/3 as low-expression level proteins.

Next, these protein sequences are stored in a fasta file, and the software CD-HIT[1] clusters these sequences with 90% sequence similarity, with the clustering results saved in a clstr file. For the clustering results, each cluster starts with “>Cluster,” followed by a cluster number. Each sequence belonging to that cluster is listed in the following lines, with one line representing one sequence. A representative sequence is specified at the end of the line with an “*,” while the remaining sequences have their similarity percentage to the representative sequence of the cluster noted at the end of their line.

After obtaining clustering results, we select reference sequences to construct the dataset from among them. The selection method for reference sequences and the resulting clustering scenarios are as follows:

**1. Each cluster contains only one sequence:** If the representative sequence of that cluster is high-expression or low-expression protein, that sequence is chosen as the reference sequence. However, if the representative sequence is a medium-expression protein, it is not selected.

**2. Each cluster contains multiple sequences:** When each cluster contains multiple sequences, the scenarios are as follows:

**(1) Each cluster contains only one type of protein:** Similar to when each cluster contains only one sequence, if the representative sequence of that cluster is a high-expression or low-expression protein, that sequence is chosen as the reference sequence. However, if the representative sequence is a medium-expression protein, it is not selected.

(2) Each cluster contains both high-expression and low-expression proteins: No reference protein is selected from that cluster.

**(3) Each cluster contains both high-expression and medium-expression proteins:** In this case, if the representative sequence of the cluster is a high-expression protein, that sequence is chosen as the reference sequence. However, if the representative sequence is a medium-expression protein, it is not selected.

**(4) Each cluster contains both low-expression and medium-expression proteins:** In this case, if the representative sequence of the cluster is a low-expression protein, that sequence is chosen as the reference sequence. However, if the representative sequence is a medium-expression protein, it is not selected.

Using the above methods, a relatively balanced dataset is obtained, containing approximately equal numbers of high-expression and low-expression proteins. For the universality of the experiment and the effectiveness of model training, only species with a remaining data volume greaterthan 1000 are selected for further analysis.

## Supplementary Method 2: Pre-training task

The input layer for the MP-TRANS network during both pre-training and fine-tuning phases consists of the addition of Token Embedding and Position Embedding (since MP-TRANS does not use the "sentence" concept, Segment Embedding is omitted compared to the BERT[4] framework). Token Embedding is the process of converting amino acids into a numerical form that the model can process, involving the concept of a "vocabulary" that includes twenty standard amino acids and some special tokens. Each token in the "vocabulary" has a corresponding embedding vector in MP-TRANS, capturing the semantic features of word fragments. For Position Embedding, since the Transformer architecture used by MP-TRANS inherently lacks the ability to process sequence order, Position Embedding assigns a unique vector to each position to provide the model with information about the word's position in the sentence, which is learned through training. The final representation of each input token in MP-TRANS is the sum of its Token Embedding and Position Embedding.

The hidden layer of MP-TRANS is a stack of multiple Transformer layers, and the output layer that follows will be designed according to the specific task of MP-TRANS, meaning the only difference between MP-TRANS's pre-training and fine-tuning network architectures lies in the output layer. During the pre-training phase, generally, the BERT architecture's pre-training requires two tasks: Masked Language Model (MaskedLM) and Next Sentence Prediction (NSP). MaskedLM is akin to a close task, while in the NSP task, BERT predicts whether two sentences appear consecutively in the original text, which is important for understanding paragraph-level semantics.

To adapt BERT for expression level prediction and mutant generation tasks, MP-TRANS omits the NSP task from BERT's pre-training, retaining only the MaskedLM task as the optimization metric for pre-training. This trains a bidirectional text encoder through a denoising objective (i.e., restoring masked amino acids), enabling it to consider the flanking sequences to the left and right of each amino acid in the input sequence. The encoder trained will produce context representations suitable for downstream tasks. In simple terms, the MaskedLM task trains the model to predict the masked portions of the sequence, thereby learning unique features of proteins.

The output layer designed for the MaskedLM task in MP-TRANS is a simple linear fully connected layer, converting the output from the Transformer layer into a dimension that matches the size of the "vocabulary." The output of this layer is processed by the softmax activation function, converting it into a probability distribution that represents the predicted Token's probability within the "vocabulary." In the MaskedLM task, this layer's primary role is to predict the tokens masked in the input sequence. For each masked token, the model attempts to predict its real token from the vocabulary. The final structure of the MP-TRANS network is shown in Figure 1(b), where the output layer is a fully connected network corresponding to each token, with an output dimension matching the "vocabulary" size. Training uses CrossEntropy Loss and the AdamWeightDecay optimizer.

## Supplementary Method 3: Pre-training Dataset and Data Processing

The pre-training data is sourced from the UniRef50[5] database provided by the UniProt website (SUZEK et al., 2015), with fasta format protein sequences obtained from the UniProt FTP site (https://ftp.uniprot.org/pub/databases/uniprot/). The protein sequences in UniRef50 originate from the UniProtKB and UniParc databases and are clustered with 50% sequence identity. This clustering feature ensures that the pre-training dataset constructed through the UniRef50 database covers the sequence space comprehensively while eliminating redundant sequences as much as possible, which also speeds up training.

MP-TRANS's self-supervised pre-training does not rely on traditional supervised learning, meaning it does not require manually pre-annotated data. In simple terms, there's no need to acquire any other information about the sequences in the UniRef50 dataset (such as the specific properties of the proteins) beforehand; the model can be trained solely based on their amino acid sequences, without needing manually constructed labels in advance.

To construct the self-supervised pre-training dataset with random masking mentioned in the previous section, we treat each protein sequence in the pre-processed UniRef50 dataset as a sentence in BERT training data, with each amino acid representing a word, i.e., a Token. For the MaskLM task processing, we randomly mask residues on each sequence with a 15% probability, with an 80% chance of using the special token “[MASK]” for random masking, a 10% chance of replacement with a random amino acid, and a 10% chance of leaving the amino acid unchanged. This training method increases the difficulty of model training, forcing the model to learn not just contextual information but also how to distinguish and correct incorrect or irrelevant information. This helps make the model more robust in downstream tasks, enabling a more comprehensive and accurate understanding of the context rather than merely relying on the presence of “[MASK]” markers.

We then add the special token “[CLS]” at the beginning of each sequence, which is used to aggregate the features of the entire sequence. At the end of each sequence, we add the special token “[SEP]”, signifying the end of the real sequence. Our model accepts a maximum protein sequence length of 1022; after adding two special tokens “[CLS]” and “[SEP]”, the maximum token sequence length defined by our model is 1024. To ensure equal length of model input token sequences, when a protein sequence is shorter than 1022, we pad the sequence to 1024 token lengths with the special token “[PAD]” after “[SEP]”; when a protein sequence length exceeds 1022, we discard the part exceeding 1022. If a sequence contains non-standard amino acids besides the 20 standard ones, we replace them with the special token “[UNK]”. The constructed "vocabulary" includes 20 amino acids and 5 special tokens, making a total of 25 "vocabulary" items.

## Supplementary Method 4: Prokaryotic Expression and Crude Enzyme Preparation of PM10868 and Its Mutants

First, the amino acid sequence of the proteins (xylanase PM10868, Cellulose Cel5A and PETase LCCICCG_I6M) were optimized according to *E. coli* codons and constructed into the prokaryotic expression vector pET-28a (+) (xylanase PM10868, Cellulose Cel5A) and pET-26b (+) (PETase LCCICCG_I6M).

The wild type and its mutant recombinant plasmids were transformed into BL21(DE3) strains, respectively (Purchased from Sangon Biotech (Shanghai) Co., Ltd.). Subsequently, white single colonies were picked, inoculated in LB liquid medium containing kanamycin sulfate and cultured overnight at 37°C, 200r/min. Take 0.5 mL of the activated bacterial liquid and inoculate it into 50 mL LB liquid medium containing kanamycin sulfate for scale-up culture (37°C, 200 r/min). When the *OD*_600_ value of the bacterial liquid reached 0.6 to 0.8, add 0.04% 1mmol/L IPTG and induce for 18 hours at 16°C, 200rpm. After induction, collect the bacterial cells by centrifugation at 12,000 rpm for 5 min. Resuspend the bacterial cells in 5 mL buffer containing 20 mM Tris-HCl (pH 7.0). The whole bacterial liquid was broken by an ultrasonic crusher under working conditions: 30W power, working for 4 seconds, stopping for 3 seconds, a total of 20 minutes of ultrasonication until the solution was clear. The supernatant obtained after centrifugation was the supernatant crude enzyme liquid, and the precipitate was resuspended in 20 mM Tris-HCl (pH 7.0) as the precipitate crude enzyme liquid. Then the samples were stored at 4°C for later use. Mix 20μL of enzyme liquid with 5μL 5x SDS-PAGE Loading Buffer, heat in a boiling water bath for 10 min, then centrifuge at 12,000 rpm for 10 min. After cooling in an ice bath for 5 min, take the same volume of the supernatant for SDS-PAGE gel electrophoresis and western blot analyses[6].

The operation of the three proteins was the same, and the experiment was repeated three times for each protein.

## Supplementary Method 5: Enzyme Activity Assay of Xylanase

The traditional 3,5-dinitrosalicylic acid (DNS) method is used to determine the enzyme activity of xylanase. In a 1 mL reaction system, add 900 µL 1% beechwood xylan, 100 µL supernatant crude enzyme liquid, and 100 µL 20 mM Tris-HCl (pH 7.0) as a blank control. React at 37°C for 10 min, then add 1.5 mL DNS to terminate the reaction and place in a boiling water bath for 5min. Take 200 µL of the sample and measure the absorbance at OD_540_, with each reaction performed in triplicate. Enzyme activity units are defined as the amount of enzyme required to release 1 μmol of reducing sugar under optimal conditions per minute. The enzyme activity calculation formula is as follows:

$$A=\left( \left( A_{1}-A_{0} \right)\times K+C_{0} \right)\times V_{1}\times n/\left( V_{2}\times t \right)$$

Where $A$ is the enzyme activity (U/mL), $A_{1}$ is the absorbance of the sample enzyme liquid (OD_540_), $A_{0}$ is the corresponding blank absorbance (OD_540_), $K$ is the slope of the p-nitrophenol standard curve, $C_{0}$ is the intercept of the p-nitrophenol standard curve. $n$ is the dilution factor, $V_{1}$ is the volume of reaction liquid (ml), $V_{2}$ is the volume of enzyme liquid (ml), and $t$ is the reaction time (min).

The next step in drawing the standard curve is to dry the xylose standards at 55°C for 6 hours, then prepare them in double-distilled water (ddH_2_O) to create concentration gradients of 0, 1, 2, 3, 4, 4.5, 5, 6, 6.5, and 7 mg/mL. Take 1 mL of each concentration gradient sample and add 1.5 mL of DNS, then place in a boiling water bath for 5 minutes, followed by centrifugation. Measure the absorbance at OD_540_ of 250 µL of the supernatant, with each reaction performed in triplicate. Plot the standard curve with the absorbance values at OD_540_ for each xylose concentration gradient on the x-axis and the xylose content on the y-axis, and fit the curve. Table S14 presents the absorbance values at OD_540_ for each xylose concentration gradient. The xylose standard curve drawn as described above is shown in Figure S7.

## Supplementary Method 6: Determination of expression level of designed cellulase and PETase

The expression levels of cellulase Cel5A, PETase LCCICCG_I6M, and three mutant variants were quantified by analyzing the grayscale intensity of Western blotting bands[7]. ImageJ[8] software was employed to process the Western blotting data, including desaturation, conversion to 8-bit format, normalization of background grayscale, and conversion to black and white. The Integrated Density parameter was set for measuring the bands, with the measurement unit as "single pixel." Specific bands were chosen for analysis, and their Integrated Density values were used for quantification of expression levels. To evaluate the relationship between HE-Value and protein expression levels, relative values for both HE-Value and protein expression levels were computed using the formula:

$$R(X)=\left( x\left( mutant \right)-x\left( wt \right) \right)/x\left( wt \right).$$

Where $x$ represents the HE-Value or expression level of the protein, $wt$ represents the corresponding value of the wild-type protein. $R(X)$ represents the relative value of $x$, where the $R(X)$ of the wild-type is defined as 0.

# Supplementary Figures

## Figure S1





Dot plot of expression level (log-treated) versus protein SRAB values and the corresponding Spearman rank correlation between expression level and versus protein SRAB values.

## Figure S2


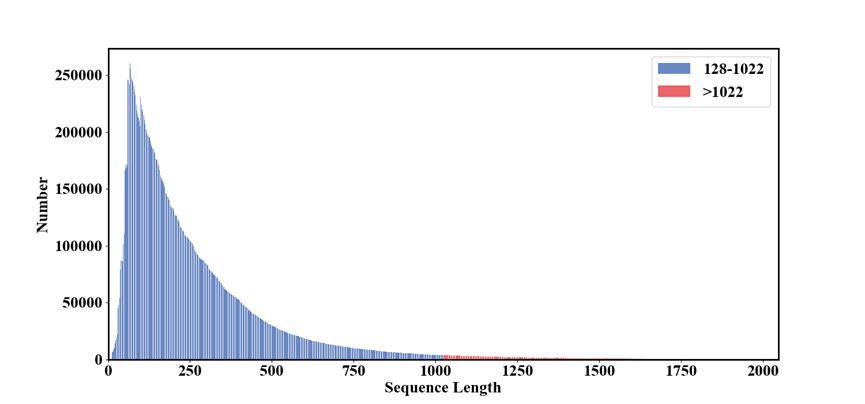


Sequence length statistics for the UniRef50 database. This figure only shows the number of sequences with sequence lengths less than 2048.

## Figure S3


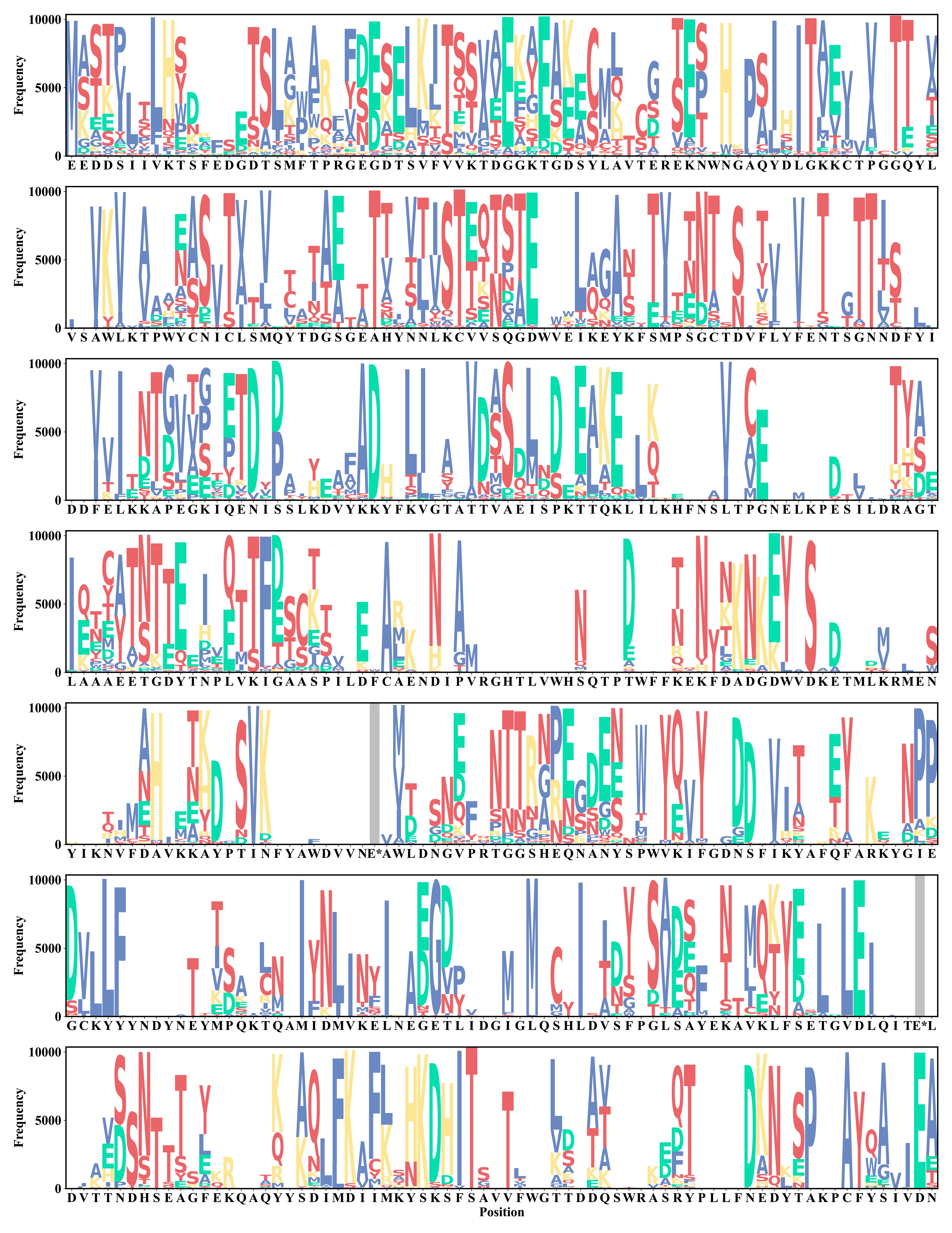


The amino acid frequency at each position across the Pm10868 mutants. The wild-type protein sequence is represented along the x-axis by the reference amino acids, the y-axis shows the frequency of observed mutations at each position. Positions with a higher number of mutations are displayed with taller letters. Each amino acid is color-coded based on its biochemical properties: nonpolar (blue), polar (red), positively charged (yellow), and negatively charged (green). The grey-shaded bars represent active sites, with positions 314E and 431E highlighted as active residues for the protein.

## Figure S4


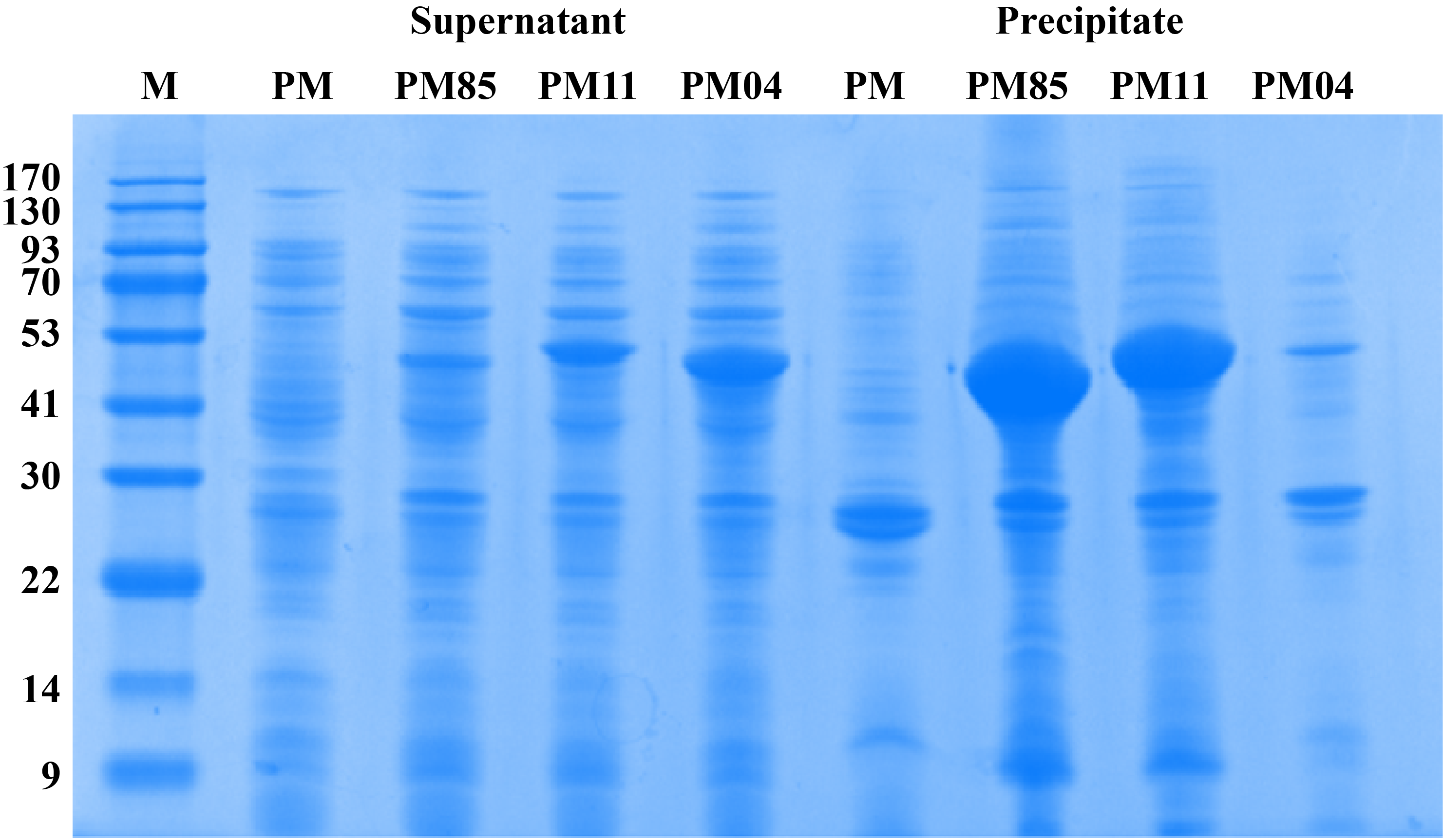


The expression of wild-type Pm10868 protein (PM) and its mutants (PM85, PM11, and PM04) in both the supernatant and precipitate fractions. The lanes labeled "Supernatant" show the soluble fractions of the proteins, while the lanes labeled "Precipitate" represent the insoluble fractions. Clear bands are visible at approximately 53 kDa, which is the expected size of the target protein. The wild-type protein (PM) is not expressed in either the supernatant or precipitate, while all the mutants show expression. mutant PM04 exhibits the highest level of soluble expression in the supernatant.

## Figure S5


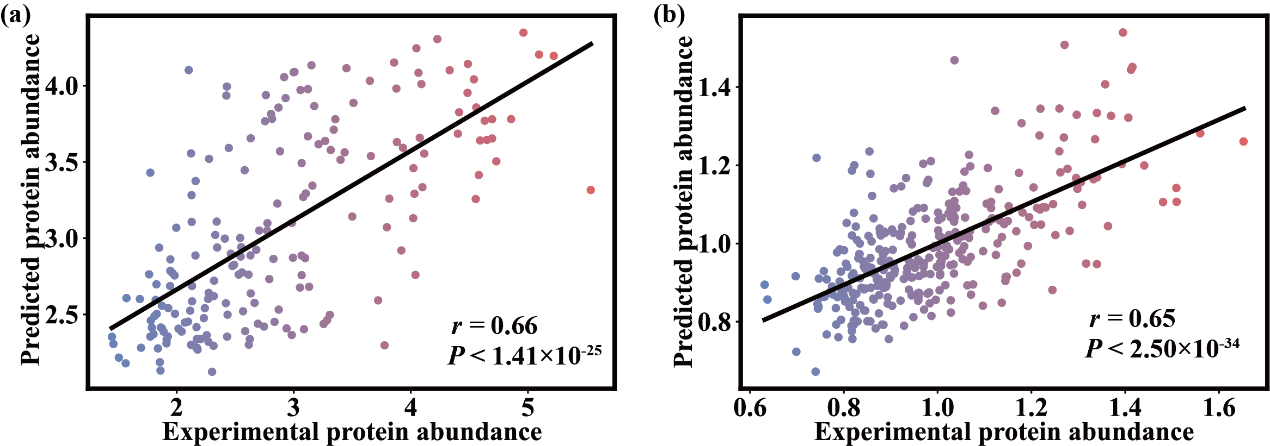


Evaluation metrics of the MPB-EXP-R model trained on two benchmark datasets. (A) Performance of the model on the Daoy medulloblastoma cell line dataset; (B) performance of the model on the S. cerevisiae mRNA translation dataset.

## Figure S6





Experimental protein abundance (log-scale) and MPB-EXP-R predicted protein abundance (log-scale).

## Figure S7


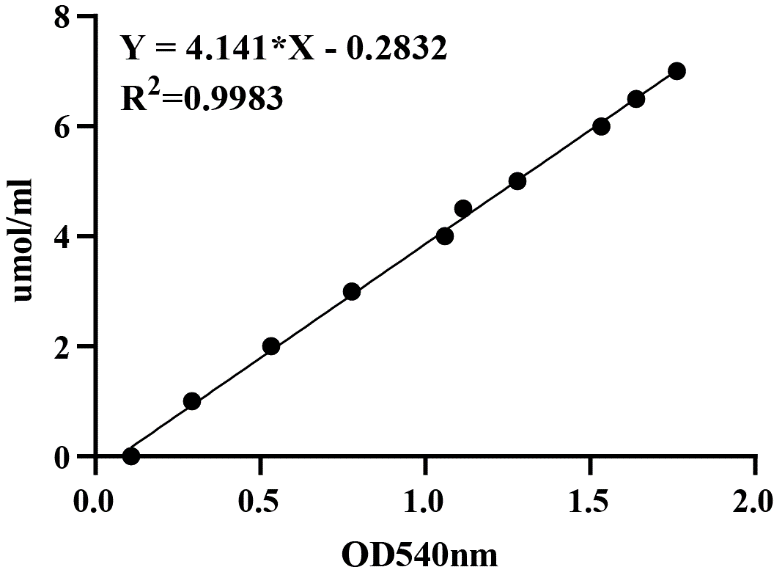


Standard curve of xylose

# Supplementary Tables

## Table S1

Network parameter of MP-TRANS

| Parameter | Value |
| --- | --- |
| hidden_size | 1024 |
| num_hidden_layers | 8 |
| num_attention_heads | 16 |
| intermediate_size | 3072 |
| hidden_dropout_prob | 0.1 |
| attention_probs_dropout_prob | 0.1 |
| initializer_range | 0.2 |
| dtype | float32 |
| compute_type | float16 |

## Table S2

Dataset information and mean evaluation results of expression models for each species on independent test sets

| taxon ID | Species or strain | Dataset | Sample size | AUC | ACC | Recall | precision | F1 | MCC |
| --- | --- | --- | --- | --- | --- | --- | --- | --- | --- |
| 547559 | *Natrialba magadii* ATCC 43099 | 547559-WHOLE_ORGANISM-integrated | 1227 | 0.73 | 0.68 | 0.67 | 0.68 | 0.67 | 0.36 |
| 64091 | *Halobacterium salinarum* NRC-1 | 64091-WHOLE_ORGANISM-integrated | 1159 | 0.89 | 0.82 | 0.84 | 0.83 | 0.83 | 0.63 |
| 880073 | *Caldithrix abyssi* DSM 13497 | 880073-PXD145877_Mueller_Nature_2020_Caldithryx_abyssi | 1387 | 0.82 | 0.75 | 0.73 | 0.73 | 0.73 | 0.49 |
| 189518 | *Leptospira interrogans* serovar Lai str. 56601 | 189518-WHOLE_ORGANISM-integrated | 1592 | 0.91 | 0.82 | 0.80 | 0.86 | 0.83 | 0.65 |
| 246200 | *Ruegeria pomeroyi* DSS-3 | 246200-WHOLE_ORGANISM-integrated | 1319 | 0.85 | 0.78 | 0.80 | 0.77 | 0.78 | 0.56 |
| 208964 | *Pseudomonas aeruginosa* PAO1 | 208964-WHOLE_ORGANISM-integrated | 2746 | 0.86 | 0.77 | 0.79 | 0.77 | 0.78 | 0.55 |
| 160488 | *Pseudomonas putida* KT2440 | 160488-PXD003826_Lidbury_Environ_Microbiol_2016_P_putida | 1357 | 0.84 | 0.77 | 0.75 | 0.78 | 0.77 | 0.54 |
| 99287 | *Salmonella enterica* subsp. enterica serovar Typhimurium str. LT2 | 99287-WHOLE_ORGANISM-integrated | 1731 | 0.87 | 0.78 | 0.80 | 0.80 | 0.80 | 0.56 |
| 511145 | *Escherichia coli* str. K-12 substr. MG1655 | 511145-WHOLE_ORGANISM-integrated | 2450 | 0.87 | 0.79 | 0.81 | 0.78 | 0.79 | 0.58 |
| 272620 | *Klebsiella pneumoniae* subsp. pneumoniae MGH 78578 | 272620-WHOLE_ORGANISM-integrated | 1542 | 0.85 | 0.77 | 0.76 | 0.78 | 0.77 | 0.54 |
| 1286170 | *Raoultella ornithinolytica* B6 | 1286170-WHOLE_ORGANISM-integrated | 1613 | 0.82 | 0.74 | 0.77 | 0.73 | 0.75 | 0.47 |
| 709991 | *Odoribacter splanchnicus* DSM 20712 | 709991-PXD145877_Mueller_Nature_2020_Odoribacter_splanchnicus | 1360 | 0.87 | 0.79 | 0.77 | 0.82 | 0.79 | 0.59 |
| 537011 | *Segatella copri* DSM 18205 | 537011-PXD145877_Mueller_Nature_2020_Prevotella_copri | 1129 | 0.84 | 0.75 | 0.73 | 0.78 | 0.75 | 0.50 |
| 411477 | *Parabacteroides merdae* ATCC 43184 | 411477-PXD145877_Mueller_Nature_2020_Parabacteroides_merdae | 1402 | 0.91 | 0.83 | 0.86 | 0.80 | 0.83 | 0.65 |
| 435591 | *Parabacteroides distasonis* ATCC 8503 | 435591-PXD145877_Mueller_Nature_2020_Parabacteroides_distasonis | 1325 | 0.87 | 0.79 | 0.80 | 0.79 | 0.79 | 0.58 |
| 435590 | *Phocaeicola vulgatus* ATCC 8482 | 435590-PXD145877_Mueller_Nature_2020_Bacteroides_vulgatus | 1377 | 0.86 | 0.77 | 0.76 | 0.78 | 0.77 | 0.55 |
| 226186 | *Bacteroides thetaiotaomicron* VPI-5482 | 226186-WHOLE_ORGANISM-integrated | 1547 | 0.86 | 0.78 | 0.79 | 0.78 | 0.78 | 0.55 |
| 272559 | *Bacteroides fragilis* NCTC 9343 | 272559-PXD145877_Mueller_Nature_2020_Bacteroides_fragilis | 1405 | 0.89 | 0.81 | 0.84 | 0.80 | 0.82 | 0.62 |
| 411479 | *Bacteroides uniformis* ATCC 8492 | 411479-PXD145877_Mueller_Nature_2020_Bacteroides_uniformis | 1451 | 0.84 | 0.75 | 0.74 | 0.76 | 0.75 | 0.50 |
| 449447 | *Microcystis aeruginosa* NIES-843 | 449447-WHOLE_ORGANISM-integrated | 2797 | 0.75 | 0.69 | 0.79 | 0.72 | 0.75 | 0.35 |
| 1140 | *Synechococcus elongatus* PCC 7942 | 1140-PXD000510_Guerreiro_Mol_Cell_Proteomics_2014_Synechococcus_elongatus | 1450 | 0.93 | 0.84 | 0.81 | 0.87 | 0.84 | 0.68 |
| 100226 | *Streptomyces coelicolor* A3(2) | 100226-WHOLE_ORGANISM-integrated | 2301 | 0.71 | 0.66 | 0.61 | 0.67 | 0.64 | 0.31 |
| 83332 | *Mycobacterium tuberculosis* H37Rv | 83332-WHOLE_ORGANISM-integrated | 2233 | 0.85 | 0.77 | 0.77 | 0.79 | 0.78 | 0.54 |
| 246196 | *Mycolicibacterium smegmatis* MC2 155 | 246196-WHOLE_ORGANISM-integrated | 1940 | 0.85 | 0.77 | 0.81 | 0.76 | 0.78 | 0.54 |
| 1280 | *Staphylococcus aureus* | 1280-158878_GPM_201408 | 1131 | 0.85 | 0.77 | 0.75 | 0.83 | 0.79 | 0.53 |
| 224308 | *Bacillus subtilis* subsp. subtilis str. 168 | 224308-WHOLE_ORGANISM-integrated | 2671 | 0.84 | 0.77 | 0.76 | 0.78 | 0.77 | 0.53 |
| 610130 | *[Clostridium] saccharolyticum* WM1 | 610130-PXD145877_Mueller_Nature_2020_Clostridium_saccharolyticum | 1396 | 0.78 | 0.72 | 0.71 | 0.74 | 0.73 | 0.43 |
| 411902 | *Enterocloster bolteae* ATCC BAA-613 | 411902-PXD145877_Mueller_Nature_2020_Clostridium_bolteae | 1410 | 0.83 | 0.76 | 0.77 | 0.76 | 0.76 | 0.51 |
| 411470 | *[Ruminococcus] gnavus* ATCC 29149 | 411470-PXD145877_Mueller_Nature_2020_Ruminococcus_gnavus | 1038 | 0.86 | 0.78 | 0.76 | 0.78 | 0.77 | 0.55 |
| 55529 | *Guillardia theta* | 55529-PXD145877_Mueller_Nature_2020_Guillardia_theta | 5889 | 0.90 | 0.82 | 0.84 | 0.81 | 0.83 | 0.64 |
| 44689 | *Dictyostelium discoideum* | 44689-WHOLE_ORGANISM-integrated | 4602 | 0.91 | 0.85 | 0.88 | 0.84 | 0.86 | 0.70 |
| 2903 | *Emiliania huxleyi* | 2903-PXD145877_Mueller_Nature_2020_Emiliania_huxleyi | 5150 | 0.88 | 0.81 | 0.82 | 0.81 | 0.81 | 0.61 |
| 5691 | *Trypanosoma brucei* | 5691-WHOLE_ORGANISM-integrated | 3670 | 0.86 | 0.78 | 0.77 | 0.78 | 0.77 | 0.56 |
| 353153 | *Trypanosoma cruzi* strain CL Brener | 353153-WHOLE_ORGANISM-integrated | 1561 | 0.84 | 0.77 | 0.80 | 0.80 | 0.80 | 0.53 |
| 2850 | *Phaeodactylum tricornutum* | 2850-PXD145877_Mueller_Nature_2020_Phaeodactylum_tricornutum | 3640 | 0.91 | 0.84 | 0.88 | 0.83 | 0.85 | 0.67 |
| 35128 | *Thalassiosira pseudonana* | 35128-PXD145877_Mueller_Nature_2020_Thalassiosira_pseudonanna | 4507 | 0.87 | 0.80 | 0.83 | 0.78 | 0.81 | 0.60 |
| 5811 | *Toxoplasma gondii* | 5811-WHOLE_ORGANISM-integrated | 1655 | 0.89 | 0.83 | 0.91 | 0.80 | 0.85 | 0.66 |
| 5833 | *Plasmodium falciparum* | 5833-WHOLE_ORGANISM-integrated | 3362 | 0.88 | 0.79 | 0.82 | 0.79 | 0.81 | 0.59 |
| 73239 | *Plasmodium yoelii* yoelii | 73239-Plasmodium-yoelii_PA_2012-9 | 1024 | 0.87 | 0.79 | 0.87 | 0.75 | 0.81 | 0.59 |
| 3055 | *Chlamydomonas reinhardtii* | 3055-WHOLE_ORGANISM-integrated | 4269 | 0.89 | 0.80 | 0.84 | 0.77 | 0.80 | 0.59 |
| 3218 | *Physcomitrium patens* | 3218-PXD013606_Physcomitrella_patens | 5911 | 0.92 | 0.84 | 0.85 | 0.82 | 0.84 | 0.69 |
| 4577 | *Zea mays* | 4577-LEAF-integrated | 7973 | 0.89 | 0.81 | 0.78 | 0.82 | 0.80 | 0.62 |
| 39947 | *Oryza sativa* Japonica Group | 39947-WHOLE_ORGANISM-integrated | 6440 | 0.87 | 0.80 | 0.82 | 0.79 | 0.80 | 0.60 |
| 4565 | *Triticum aestivum* | 4565-LEAF-integrated | 3273 | 0.69 | 0.64 | 0.54 | 0.65 | 0.59 | 0.28 |
| 4513 | *Hordeum vulgare* | 4513-ENDOSPERM-integrated | 2056 | 0.82 | 0.75 | 0.79 | 0.73 | 0.76 | 0.50 |
| 4097 | *Nicotiana tabacum* | 4097-LEAF-integrated | 3177 | 0.89 | 0.82 | 0.81 | 0.81 | 0.81 | 0.64 |
| 4081 | *Solanum lycopersicum* | 4081-PXD012810_McWhite_Cell_2020_Tomato_green | 4873 | 0.70 | 0.64 | 0.60 | 0.67 | 0.63 | 0.29 |
| 4113 | *Solanum tuberosum* | 4113-LEAF-integrated | 4302 | 0.89 | 0.82 | 0.85 | 0.80 | 0.82 | 0.63 |
| 29760 | *Vitis vinifera* | 29760-SHOOT-integrated | 1385 | 0.89 | 0.80 | 0.81 | 0.76 | 0.78 | 0.60 |
| 3880 | *Medicago truncatula* | 3880-PXD002692_flower | 8378 | 0.89 | 0.82 | 0.82 | 0.83 | 0.82 | 0.64 |
| 3847 | *Glycine max* | 3847-SHOOT-integrated | 1664 | 0.78 | 0.71 | 0.77 | 0.69 | 0.72 | 0.42 |
| 2711 | *Citrus sinensis* | 2711-PXD008366_leaf | 1882 | 0.87 | 0.77 | 0.79 | 0.76 | 0.77 | 0.54 |
| 3635 | *Gossypium hirsutum* | 3635-LEAF-integrated | 4341 | 0.87 | 0.80 | 0.77 | 0.76 | 0.76 | 0.59 |
| 3702 | *Arabidopsis thaliana* | 3702-WHOLE_ORGANISM-integrated | 13168 | 0.94 | 0.87 | 0.88 | 0.86 | 0.87 | 0.74 |
| 3708 | *Brassica napus* | 3708-PXD005787_Geng_Front_Mol_Biosci_2017_Bnapus_guard_cell | 2949 | 0.83 | 0.78 | 0.85 | 0.76 | 0.80 | 0.56 |
| 214684 | *Cryptococcus neoformans* JEC21 | 214684-WHOLE_ORGANISM-integrated | 1606 | 0.82 | 0.78 | 0.87 | 0.76 | 0.81 | 0.55 |
| 4896 | *Schizosaccharomyces pombe* | 4896-WHOLE_ORGANISM-integrated | 3010 | 0.88 | 0.81 | 0.83 | 0.81 | 0.82 | 0.62 |
| 5507 | *Fusarium oxysporum* | 5507-PXD145877_Mueller_Nature_2020_Fusarium_oxisporum | 2805 | 0.81 | 0.73 | 0.78 | 0.70 | 0.74 | 0.48 |
| 5141 | *Neurospora crassa* | 5141-PXD145877_Mueller_Nature_2020_Neurospora_crassa | 3419 | 0.90 | 0.82 | 0.88 | 0.81 | 0.84 | 0.64 |
| 5476 | *Candida albicans* | 5476-Candida-albicans_PA_2015-2 | 2586 | 0.91 | 0.84 | 0.87 | 0.82 | 0.85 | 0.69 |
| 284590 | *Kluyveromyces lactis* NRRL Y-1140 | 284590-WHOLE_ORGANISM-integrated | 1932 | 0.93 | 0.85 | 0.88 | 0.84 | 0.86 | 0.71 |
| 4932 | *Saccharomyces cerevisiae* | 4932-WHOLE_ORGANISM-integrated | 4042 | 0.93 | 0.87 | 0.91 | 0.85 | 0.88 | 0.73 |
| 6239 | *Caenorhabditis elegans* | 6239-WHOLE_ORGANISM-integrated | 8396 | 0.92 | 0.84 | 0.88 | 0.82 | 0.85 | 0.69 |
| 6945 | *Ixodes scapularis* | 6945-PXD002181_Villar_Mol_Cell_Proteomics_2015_tick_proteome_uninfected_NSAF | 2563 | 0.75 | 0.69 | 0.72 | 0.67 | 0.70 | 0.38 |
| 7460 | *Apis mellifera* | 7460-WHOLE_ORGANISM-integrated | 2271 | 0.86 | 0.80 | 0.81 | 0.79 | 0.80 | 0.59 |
| 7227 | *Drosophila melanogaster* | 7227-WHOLE_ORGANISM-integrated | 8643 | 0.91 | 0.83 | 0.85 | 0.81 | 0.83 | 0.65 |
| 7165 | *Anopheles gambiae* | 7165-GPM_201408 | 2729 | 0.84 | 0.76 | 0.77 | 0.73 | 0.75 | 0.51 |
| 7159 | *Aedes aegypti* | 7159-PXD020408_susceptible_reference_strain_new_orleans_midgut | 2430 | 0.82 | 0.77 | 0.80 | 0.78 | 0.79 | 0.53 |
| 7955 | *Danio rerio* | 7955-WHOLE_ORGANISM-integrated | 8854 | 0.94 | 0.86 | 0.91 | 0.83 | 0.87 | 0.73 |
| 30732 | *Oryzias melastigma* | 30732-PXD145877_Mueller_Nature_2020_Oryzias_melastigma | 4122 | 0.90 | 0.82 | 0.88 | 0.80 | 0.84 | 0.65 |
| 8030 | *Salmo salar* | 8030-LIVER-integrated | 1620 | 0.83 | 0.77 | 0.75 | 0.76 | 0.76 | 0.53 |
| 8022 | *Oncorhynchus mykiss* | 8022-PXD010186_rainbow_trout_liver | 3045 | 0.83 | 0.76 | 0.78 | 0.76 | 0.77 | 0.52 |
| 8364 | *Xenopus tropicalis* | 8364-GPM_2012_09_Xenopus_tropicalis | 1227 | 0.83 | 0.77 | 0.73 | 0.76 | 0.74 | 0.53 |
| 8355 | *Xenopus laevis* | 8355-EMBRYO-integrated | 1535 | 0.85 | 0.76 | 0.80 | 0.74 | 0.77 | 0.53 |
| 9031 | *Gallus gallus* | 9031-WHOLE_ORGANISM-integrated | 7962 | 0.89 | 0.82 | 0.84 | 0.81 | 0.82 | 0.64 |
| 9612 | *Canis lupus* | 9612-PXD145877_Mueller_Nature_2020_Canis_lupus_CCL-34_kidney | 4779 | 0.88 | 0.80 | 0.82 | 0.79 | 0.81 | 0.60 |
| 9615 | *Canis lupus* (familiaris) | 9615-GPM_2012_09_Canis_familiaris | 2590 | 0.90 | 0.82 | 0.85 | 0.78 | 0.81 | 0.65 |
| 9796 | *Equus caballus* | 9796-PA_201304 | 1969 | 0.85 | 0.76 | 0.81 | 0.71 | 0.76 | 0.53 |
| 9823 | *Sus scrofa* | 9823-WHOLE_ORGANISM-integrated | 5061 | 0.87 | 0.79 | 0.84 | 0.77 | 0.80 | 0.59 |
| 9913 | *Bos taurus* | 9913-WHOLE_ORGANISM-integrated | 6869 | 0.91 | 0.84 | 0.84 | 0.84 | 0.84 | 0.69 |
| 89462 | *Bubalus bubalis* | 89462-PXD003859_sperm | 1597 | 0.76 | 0.70 | 0.66 | 0.71 | 0.68 | 0.39 |
| 9544 | *Macaca mulatta* | 9544-LIVER-integrated | 5730 | 0.82 | 0.74 | 0.75 | 0.72 | 0.74 | 0.48 |
| 9598 | *Pan troglodytes* | 9598-Chimp_iBAQ_Khan_2013 | 1909 | 0.84 | 0.77 | 0.83 | 0.74 | 0.78 | 0.55 |
| 9606 | *Homo sapiens* | 9606-WHOLE_ORGANISM-integrated | 12201 | 0.92 | 0.85 | 0.87 | 0.84 | 0.86 | 0.70 |
| 9986 | *Oryctolagus cuniculus* | 9986-PXD016866_rabbit_spleen | 3686 | 0.85 | 0.77 | 0.79 | 0.76 | 0.77 | 0.53 |
| 10029 | *Cricetulus griseus* | 10029-PXD145877_Mueller_Nature_2020_Cricetulus_griseus_CHO | 3926 | 0.86 | 0.78 | 0.80 | 0.76 | 0.78 | 0.56 |
| 10090 | *Mus musculus* | 10090-WHOLE_ORGANISM-integrated | 11982 | 0.92 | 0.84 | 0.88 | 0.83 | 0.85 | 0.68 |
| 10116 | *Rattus norvegicus* | 10116-WHOLE_ORGANISM-integrated | 10091 | 0.91 | 0.84 | 0.87 | 0.81 | 0.84 | 0.67 |

## Table S3 Mann-Whitney U significance of the prediction accuracy of the two transfer learning methods and the traditional machine learning method.

| model | ESM+BiLSTM | MPB-EXP |
| --- | --- | --- |
| SVM | 0.0000 *** | 0.0000 *** |
| KNN | 0.0000 *** | 0.0000 *** |
| GBDT | 0.0000 *** | 0.0000 *** |
| CNN | 0.0310 * | 0.0028 ** |
| BiLSTM | 0.6180 - | 0.0160 * |
| non-pre-trained | 0.7873 - | 0.0202 * |

For each model, the calculated significance data were a total of 15 models obtained from the cross-validation of three species, *E. coli*, *Bacillus subtilis* and *Saccharomyces cerevisia*e. "*" signifies *P* < 0.05, "**" denotes *P* < 0.01, and "***" represents *P* < 0.001, reflecting varying levels of statistical significance.

## Table S4 The amino acid sequences of Pm10868 and its three mutants

| Protein | Sequence |
| --- | --- |
| Pm10868 | EEDDSIIVKTSFEDGDTSMFTPRGEGDTSVFVVKTDGGKTGDSYLAVTEREKNWNGAQYDLGKKCTPGGQYLVSAWLKTPWYCNICLSMQYTDGSGEAHYNNLKCVVSQGDWVEIKEYKFSMPSGCTDVFLYFENTSGNNDFYIDDFELKKAPEGKIQENISSLKDVYKKYFKVGTATTVAEISPKTTQKLILKHFNSLTPGNELKPESILDRAGTLAAAEETGDYTNPLVKIGAASPILDFCAENDIPVRGHTLVWHSQTPTWFFKEKFDADGDWVDKETMLKRMENYIKNVFDAVKKAYPTINFYAWDVVNEAWLDNGVPRTGGSHEQNANYSPWVKIFGDNSFIKYAFQFARKYGIEGCKYYYNDYNEYMPQKTQAMIDMVKELNEGETLIDGIGLQSHLDVSFPGLSAYEKAVKLFSETGVDLQITELDVTTNDHSEAGFEKQAQYYSDIMDIIMKYSKSFSAVVFWGTTDDQSWRASRYPLLFNEDYTAKPCFYSIVDNIE |
| Pm10868_85 | MADDSIIVKTSFEDGDTSMFTPRGEGDTSVLVVKTDGGKEGDSYLAVTEREKNWNGAQYDLTKKCTPGGTYLVSAWLKTPWYCNICLSMQYTDGSGEAHYNNLKCVVSQGEWVEIQEYKFSMPSNCTDVFLYFENTGGNNDFYIDDFELKKAPEGKIQEDISSLKDVYKKYFKVGAATTVAEISPKTTQKLILKHFNSLTPGNELKPESILDRAGTLAAAEETGDYTNPLVKFGAASPILDFCAENNIPVRGHTLVWHSQTPTWFFKEKFKKDGEWVDKETMLKRMENYIKNVFDAVKKAYPTIDFYAWDVVNEAWLDNGVPRTGGSHEQNANYSPWVKIFGDNSFIKYAFQFARKYNIEGCKYYYNDYNEYMPQKTQAMIDMVKELNEGETYIDGIGLQSHLDVSFPSLSAYEEAVKLFSETGVDLQITELDVTTNDHSEAGFEKQAQRYSDIMKIIMKYSKSFSSVTFWGTTDDQSWRASRYPLLFNEDYTAKPCFYSIVDNIE |
| Pm10868_11 | EEDDSIIVKTSFEDGDTSKFTPRGEGDESVFVVKTDGGKTGDSYKAVTEREKNWNKAQYDLGKKCTPGGQYLVSAWLKTPWYCNICLSVQYTDGSVETHLNNLKCVKSQGDWVEIKEAKFSMPSGCTDVFLYFENTSGNNDFYIDDFELKKAPEGKIQENISSLKDVYKKYFKDFTATTVEEISPKTTQKLILKHFNSLTPGNELKPESILDRHDTLAAAEETGDYTNPEVKIGAASPILDFCAENDIAVRGHTLVWHSQTPDWFFKEKFDADGEWVDKETMLKRMENYIKNVFDAVKKHYPTINFYAWDVVNEAWLDNGVPRTGGSHEENANESPWVKIFGDNSFIKYAFQYARKYGIEGCKYYYNDYNEYMPQKTQAMYDMVKEINEGETLIDGIGLQSHLDISFPGLDAYEKAVKLFDETGVDLQITELDVTTNDHSEAGFEKQAQYYSDIMDIIMKYSKSFSAVVFWGTTDDQSWRASRYPLLFNEDYTAKPAFYSIVDNIE |
| Pm10868_04 | EEDDSIIVHTSFEDGDTSMFAPRGEGDTSVFVSKTDGEKTKDSYLAVTEREKNWNGAQYDLGKKCTPGGQYLVSAKLKTPWYCNICLSMQVTDGEGEAHYNNLKCEVSQGDWVEIKEYKFSMPSGCTDVFLYFENTSGNNDFYIDDFELKKAGEGKIQENISSLKDVYKKYFKVGTATTVAEISPKTTQKLILKHFNSLTPGNELKPESILDRAATLAAAEETGDYTNPLVKIGAATPILDFCAENDIPVRGHTLVWHSQTPTWFFKEKFDKDGDWVSKETMLKRMENYIKNVFDAVKKAYPDINFYAWDVVNEAWLDNGVPRTGGSHEQNAEYSPWVKIFGDNSFIKYAFQFARKYGIEGCKLYYNDYNEYMPQKTQAMIDMVKELNEGETLIDGIGLQSHLDVSFPGADAYEKAVKLFSETGVDLQITELDVTTNDHSEAGFEKQAQYYSDIMKIIMKYSKSFSAVVFWGLTDDKSWRASDYPLLFDKDYKAKPCFQSIVDNIE |

## Table S5 Pm10868 and its three mutant *E. coli* codon-optimised DNA sequences

| Protein | Sequence |
| --- | --- |
| Pm10868 | GAAGAAGATGATAGTATCATCGTGAAAACCAGTTTTGAAGATGGTGACACCAGTATGTTTACCCCGCGTGGCGAAGGTGACACCTCAGTTTTTGTTGTGAAAACCGATGGTGGTAAAACCGGTGACAGTTATCTGGCAGTGACCGAACGTGAAAAGAATTGGAATGGTGCCCAGTATGATCTGGGCAAAAAATGTACCCCGGGTGGTCAGTATCTGGTTAGCGCATGGCTGAAAACCCCGTGGTATTGCAATATTTGTCTGAGTATGCAGTATACCGATGGCAGCGGTGAAGCCCATTATAATAATCTGAAATGTGTTGTTAGCCAGGGTGACTGGGTTGAAATTAAGGAATATAAATTCAGCATGCCGAGCGGCTGTACCGATGTTTTTCTGTATTTTGAAAACACCAGTGGCAATAATGATTTTTATATCGATGACTTCGAGCTGAAAAAAGCACCGGAAGGCAAAATTCAGGAAAATATTAGCAGTCTGAAGGATGTTTATAAGAAATATTTCAAGGTGGGCACCGCCACCACCGTTGCAGAAATTAGCCCGAAAACCACCCAGAAACTGATTCTGAAACATTTTAATAGCCTGACCCCGGGTAATGAACTGAAACCGGAAAGCATTCTGGATCGTGCAGGTACCCTGGCAGCCGCCGAAGAAACCGGCGATTATACCAATCCGCTGGTTAAAATTGGTGCAGCAAGTCCGATTCTGGATTTTTGCGCAGAAAATGATATTCCGGTTCGTGGTCATACCCTGGTGTGGCATAGTCAGACCCCGACCTGGTTTTTCAAAGAAAAATTTGATGCCGATGGTGACTGGGTGGATAAAGAAACCATGCTGAAACGTATGGAAAATTATATTAAGAACGTGTTCGACGCAGTGAAAAAAGCCTATCCGACCATTAATTTTTACGCATGGGATGTTGTTAACGAAGCATGGCTGGATAATGGTGTTCCGCGCACCGGCGGCAGCCATGAACAGAATGCAAATTATAGCCCGTGGGTGAAAATTTTTGGCGATAATAGTTTTATCAAGTACGCCTTTCAGTTTGCACGCAAATATGGCATTGAAGGCTGTAAATATTACTATAATGACTACAACGAGTACATGCCGCAGAAAACCCAGGCCATGATTGATATGGTTAAAGAACTGAATGAGGGCGAAACCCTGATTGATGGCATTGGCCTGCAGAGCCATCTGGATGTGAGCTTTCCGGGCCTGAGCGCCTATGAAAAAGCCGTTAAACTGTTTAGTGAAACCGGTGTTGATCTGCAGATTACCGAACTGGATGTTACCACCAATGATCATAGCGAAGCCGGCTTTGAAAAACAGGCACAGTATTATAGTGATATTATGGATATCATCATGAAGTACAGTAAGAGTTTTAGTGCCGTGGTGTTTTGGGGTACCACCGATGATCAGAGTTGGCGTGCAAGTCGTTATCCGCTGCTGTTTAATGAAGATTATACCGCCAAACCGTGCTTTTATAGTATTGTTGATAACATTGAGTAA |
| Pm10868_85 | ATGGCAGATGATAGCATTATTGTGAAAACCAGTTTTGAAGATGGTGACACCAGCATGTTTACCCCGCGCGGCGAAGGCGATACCAGTGTGCTGGTTGTTAAAACCGATGGTGGCAAAGAAGGCGATAGCTATCTGGCCGTTACCGAACGCGAAAAGAATTGGAATGGCGCCCAGTATGATCTGACCAAAAAATGTACCCCGGGCGGCACCTATCTGGTGAGTGCCTGGCTGAAAACCCCGTGGTATTGTAATATTTGTCTGAGCATGCAGTATACCGATGGCAGTGGTGAAGCCCATTATAATAATCTGAAATGTGTGGTTAGCCAGGGTGAATGGGTTGAAATTCAGGAATATAAATTCAGCATGCCGAGTAATTGCACCGATGTTTTTCTGTATTTTGAAAATACCGGCGGTAATAATGATTTTTATATCGATGATTTCGAGCTGAAAAAGGCACCGGAAGGTAAAATTCAGGAAGATATTAGCAGCCTGAAAGATGTTTATAAAAAGTATTTCAAGGTGGGTGCCGCCACCACCGTGGCCGAAATTAGTCCGAAAACCACCCAGAAACTGATTCTGAAACATTTTAATAGCCTGACCCCGGGCAATGAACTGAAACCGGAAAGCATTCTGGATCGCGCCGGTACCCTGGCAGCAGCCGAAGAAACCGGTGACTATACCAATCCGCTGGTTAAATTTGGCGCAGCCAGTCCGATTCTGGATTTTTGTGCCGAAAATAATATCCCGGTGCGCGGTCATACCCTGGTGTGGCATAGCCAGACCCCGACCTGGTTTTTCAAAGAAAAATTCAAAAAGGACGGCGAATGGGTTGATAAAGAAACCATGCTGAAACGTATGGAAAATTATATTAAGAACGTGTTCGACGCCGTTAAAAAAGCCTATCCGACCATTGATTTTTATGCATGGGATGTGGTGAATGAAGCCTGGCTGGATAATGGCGTTCCGCGTACCGGCGGCAGCCATGAACAGAATGCCAATTATAGCCCGTGGGTTAAAATTTTTGGTGACAATAGCTTTATCAAGTACGCCTTTCAGTTTGCCCGTAAATATAATATTGAGGGTTGCAAATACTACTACAATGATTATAACGAGTACATGCCGCAGAAAACCCAGGCAATGATTGATATGGTTAAAGAACTGAATGAGGGTGAAACCTATATTGATGGCATTGGTCTGCAGAGTCATCTGGATGTGAGTTTTCCGAGCCTGAGCGCCTATGAAGAAGCAGTGAAACTGTTTAGCGAAACCGGTGTTGATCTGCAGATTACCGAACTGGATGTGACCACCAATGATCATAGCGAAGCAGGCTTTGAAAAACAGGCACAGCGCTATAGTGATATTATGAAAATTATCATGAAGTACAGCAAGAGCTTTAGTAGCGTTACCTTTTGGGGTACCACCGATGATCAGAGTTGGCGTGCAAGTCGCTATCCGCTGCTGTTTAATGAAGATTATACCGCCAAACCGTGTTTTTATAGTATTGTTGATAACATCGAG |
| Pm10868_11 | GAAGAAGATGATAGCATTATCGTGAAAACCAGCTTTGAAGATGGCGATACCAGTAAATTCACTCCGCGCGGCGAAGGCGATGAAAGTGTGTTTGTTGTTAAAACCGATGGCGGTAAAACCGGTGACAGCTATAAAGCCGTGACCGAACGCGAAAAGAATTGGAATAAGGCCCAGTATGATCTGGGTAAAAAATGTACCCCGGGTGGCCAGTATCTGGTGAGCGCCTGGCTGAAAACCCCGTGGTATTGCAATATTTGCCTGAGCGTGCAGTATACCGATGGCAGCGTGGAAACCCATCTGAATAATCTGAAATGTGTTAAAAGCCAGGGTGACTGGGTTGAAATTAAGGAAGCAAAATTTTCAATGCCGAGTGGCTGCACCGATGTTTTTCTGTATTTTGAAAATACCAGCGGCAATAATGATTTTTATATCGATGATTTCGAGCTGAAAAAGGCACCGGAAGGCAAAATTCAGGAAAATATTAGCAGCCTGAAAGATGTGTATAAAAAATATTTCAAGGACTTCACCGCCACCACCGTGGAAGAAATTAGCCCGAAAACCACCCAGAAACTGATTCTGAAACATTTTAATAGCCTGACCCCGGGTAATGAACTGAAACCGGAAAGTATTCTGGATCGTCATGATACCCTGGCCGCAGCAGAAGAAACCGGCGATTATACCAATCCGGAAGTGAAAATTGGCGCCGCCAGTCCGATTCTGGATTTTTGTGCAGAAAATGATATCGCCGTGCGTGGCCATACCCTGGTGTGGCATAGCCAGACCCCGGATTGGTTTTTCAAAGAAAAATTTGATGCGGATGGTGAATGGGTTGATAAAGAAACCATGCTGAAACGCATGGAAAATTATATTAAGAACGTGTTTGACGCAGTGAAAAAACATTATCCGACCATTAATTTCTACGCCTGGGATGTTGTGAATGAAGCCTGGCTGGATAATGGCGTGCCGCGCACCGGCGGTAGTCATGAAGAAAATGCCAATGAAAGTCCGTGGGTGAAAATTTTTGGCGATAATAGCTTTATCAAGTACGCCTTTCAGTATGCCCGTAAATATGGTATTGAAGGCTGTAAATATTACTACAATGACTATAACGAGTACATGCCGCAGAAAACCCAGGCCATGTATGATATGGTGAAAGAAATTAATGAGGGTGAAACCCTGATTGATGGTATTGGTCTGCAGAGTCATCTGGATATTAGCTTTCCGGGCCTGGATGCCTATGAAAAAGCAGTTAAACTGTTTGATGAGACCGGTGTGGATCTGCAGATTACCGAACTGGATGTGACCACCAATGATCATAGTGAAGCCGGTTTTGAAAAACAGGCACAGTATTATAGCGATATTATGGATATTATCATGAAGTACAGCAAGAGTTTTAGCGCCGTGGTGTTTTGGGGTACCACCGATGATCAGAGTTGGCGTGCAAGCCGTTATCCGCTGCTGTTTAATGAAGATTATACCGCCAAACCGGCCTTTTATAGTATTGTTGATAATATTGAG |
| Pm10868_04 | GAAGAAGATGATAGTATCATCGTGCATACCAGCTTTGAAGATGGTGACACCAGCATGTTTGCACCGCGCGGTGAAGGTGACACCAGTGTTTTTGTTAGTAAAACCGATGGCGAAAAAACCAAAGATAGTTATCTGGCAGTGACCGAACGTGAAAAGAATTGGAATGGCGCACAGTATGATCTGGGTAAAAAATGTACCCCGGGCGGTCAGTATCTGGTTAGTGCAAAACTGAAAACCCCGTGGTATTGCAATATTTGCCTGAGCATGCAGGTTACCGATGGTGAAGGCGAAGCCCATTATAATAATCTGAAATGTGAAGTTAGCCAGGGCGATTGGGTTGAAATTAAGGAATATAAATTCAGCATGCCGAGCGGTTGTACCGATGTTTTTCTGTATTTTGAAAACACCAGTGGCAATAATGATTTTTATATCGATGACTTCGAGCTGAAAAAAGCAGGTGAAGGTAAAATTCAGGAAAATATTAGTAGCCTGAAGGATGTGTATAAAAAATATTTCAAGGTGGGTACCGCAACCACCGTTGCAGAAATTAGCCCGAAAACCACCCAGAAACTGATTCTGAAACATTTTAATAGCCTGACCCCGGGCAATGAACTGAAACCGGAAAGCATTCTGGATCGTGCAGCCACCCTGGCCGCAGCAGAAGAAACCGGCGATTATACCAATCCGCTGGTTAAAATTGGTGCAGCCACCCCGATTCTGGATTTTTGCGCAGAAAATGATATTCCGGTTCGTGGCCATACCCTGGTTTGGCATAGTCAGACCCCGACCTGGTTTTTCAAAGAAAAATTTGATAAGGACGGCGATTGGGTGAGTAAAGAAACCATGCTGAAACGCATGGAAAATTATATTAAGAACGTGTTTGACGCCGTTAAAAAAGCATATCCGGATATTAATTTCTACGCCTGGGATGTGGTTAATGAAGCATGGCTGGATAATGGCGTGCCGCGTACCGGTGGTAGCCATGAACAGAATGCAGAATATAGTCCGTGGGTTAAAATTTTTGGTGACAATAGCTTTATCAAGTACGCATTTCAGTTTGCCCGTAAATATGGTATTGAAGGCTGTAAACTGTATTATAATGACTATAACGAGTACATGCCGCAGAAAACCCAGGCAATGATTGATATGGTGAAAGAACTGAATGAAGGCGAAACCCTGATTGATGGTATTGGCCTGCAGAGTCATCTGGATGTTAGCTTTCCGGGCGCCGATGCATACGAAAAAGCAGTTAAACTGTTTAGCGAAACCGGCGTTGATCTGCAGATTACCGAACTGGATGTGACCACCAATGATCATAGCGAAGCCGGCTTTGAAAAACAGGCCCAGTATTATAGCGATATTATGAAAATTATCATGAAGTACAGCAAGAGCTTTAGTGCAGTTGTGTTTTGGGGCCTGACCGATGATAAAAGTTGGCGTGCAAGCGATTATCCGCTGCTGTTTGATAAAGATTATAAAGCCAAACCGTGCTTTCAGAGCATTGTTGATAATATTGAA |

## Table S6 The values of Predicted high-level expression propensity in *E. coli* of the cellulase Cel5A, PETase LCCICCG_I6M and their mutants.

| id | Predicted high-level expression propensity in *E. coli* |
| --- | --- |
| Cel5A_WT | 0.548 |
| Cel5A_39 | 0.998 |
| Cel5A_22 | 0.683 |
| Cel5A_79 | 0.659 |
| I6M_WT | 0.352 |
| I6M_75 | 0.476 |
| I6M_69 | 0.459 |
| I6M_10 | 0.456 |

Note: Predicted high-level expression propensity, ranging from 0 to 1, is determined by the classification model.

## Table S7 The amino acid sequences of Cel5A and its three mutants

| Protein | Sequence |
| --- | --- |
| Cel5A | GPWQQCGGIGWQGSTDCVSGYHCVYQNDWYSQCVPGAASTTLQTSTTSRPTATSTAPPSSTTSPSKGKLKWLGSNESGAEFGEGNYPGLWGKHFIFPSTSAIQTLINDGYNIFRIDFSMERLVPNQLTSSFDQGYLRNLTEVVNFVTNAGKYAVLDPHNYGRYYGNIITDTNAFRTFWTNLAKQFASNSLVIFDTNNEYNTMDQTLVLNLNQAAIDGIRAAGATSQYIFVEGNAWSGAWSWNTTNTNMAALTDPQNKIVYEMHQYLDSDSSGTHAECVSSTIGAQRVVGATQWLRANGKLGVLGEFAGGANAVCQQAVTGLLDHLQDNSDVWLGALWWAAGPWWGDYMYSFEPPSGTGYVNYNSILKKYLP |
| Cel5A_39 | GPWQQCGGIGWQGSTDCVSGYHCVYQNDWYSQCVPGAASTTLQTSTTSRPTATSTAPPSSTTSPSKGKLKWLGSNESGAEFGEGNYPGLWGKHFTFPSTSTIQTLINDGYNIFRIDFSMERLVPNQLTSSFDQGYLRNLTEVVNFVTNAGAYAVLDPHNYGRYYGNIITDTNAFRTFWTNLAKQFASNSLVIFDTNNEYNSMDQTLVLNLNQAAINGIRAAGATSQYIFVEGNAWSGAWSWNTTNTNMAALTDPQNKIVYEMHQYLDSDSSGTHAECVSSTIGAQRVVGATQWLRANGKLGVLGEFAGGANAVCQQAVTGLLDHLQSNSDVWLGALWWAAGPWWGDYMYSFEPPSGTGYVNYNSILKKYLP |
| Cel5A_22 | GPWQQCGGIGWQGPTDCVSGYHCVYQNDWYSQCVPGAASTTLQTSTTSRPTATSTAPPSSTTSPPKGKLKWLGSNESGAEFGEGNYPGLWGKDFIFPSTSAIQTLINDGYNIFRIDFSMERLVPNQLTSSFDQGYLRNLTEVVNFVTNAGAYAVLDPHNYGRYYGNIITDTNAFRTFWTNLAKQFASNSLVIFDTNNEYNTMDQTLVLNLNQAAIDGIRAAGATSQYIFVEGNAWSGAWSWNTTNTNMAALTDPQNKIVYEMHQYLDSDSSGTHAECVSSTIGAQRVVGATQWLRANGKLGVLGEFAGGANAVCQQAVTGLLDHLQDNSDVWLGALWWAAGPWWGTYMYSFEPPSGTGYTNYNSILKKYAP |
| Cel5A_79 | GPWQQCGGIGWQGSTDCVAPYTCVYQNDWYSQCVPGAASTTLQTSTTSAPTATSTAPPSSTTSPSKGKLKWLGSNESGAEFGEGNYPGLWGKHFIFPSTSAIQTLINDGYNIFRIDFSMERLVPNQLTSSFDQGYLRNLTEVVNFVTNAGKYAVLDPHNYGRYYGNIITDTNAFRTFWTNLAKQFASNSLVIFDTNNEYNTMDQTLVLNLNQAAIDGIRAAGATSQYIFVEGNAWSGAWSWNTTNTNMAALTDPQNKIVYEMHQYLDSDSSGTHAECVSSTIGAQRVVGATQWLRANNKLGVLGEFAGGANAVCQQAVTGLLDYMQDNSDVWLGALWWAAGPWWGDYMYSFEPPSGTGYVNYNSILKKYLP |

Table S8 Cel5A and its three mutant *E. coli* codon-optimized DNA sequences

| Protein | Sequence |
| --- | --- |
| Cel5A_WT | GGTCCTTGGCAGCAGTGTGGCGGCATTGGTTGGCAGGGTAGTACCGATTGCGTTAGCGGTTATCATTGCGTGTATCAGAATGATTGGTATAGCCAGTGTGTTCCGGGCGCCGCAAGTACCACCCTGCAGACCAGTACCACCAGTCGCCCGACCGCAACCAGTACCGCACCGCCTAGCAGTACCACCTCACCGAGCAAAGGCAAACTGAAATGGCTGGGTAGCAATGAAAGCGGTGCAGAATTTGGTGAAGGCAATTATCCGGGCCTGTGGGGCAAACATTTTATTTTTCCGAGCACCAGTGCCATTCAGACCCTGATTAATGATGGCTATAATATTTTCCGCATCGATTTTAGCATGGAACGTCTGGTGCCGAATCAGCTGACCAGTAGTTTTGATCAGGGCTATCTGCGCAATCTGACCGAAGTGGTTAATTTTGTGACCAATGCCGGTAAATATGCAGTGCTGGATCCGCATAATTATGGCCGCTATTATGGCAATATTATTACCGATACCAACGCATTTCGTACCTTTTGGACCAATCTGGCAAAACAGTTTGCCAGTAATAGTCTGGTTATTTTTGATACCAACAACGAATATAACACCATGGATCAGACCCTGGTTCTGAATCTGAATCAGGCCGCCATTGATGGTATTCGTGCAGCAGGCGCAACCAGCCAGTATATTTTTGTTGAAGGCAATGCCTGGAGTGGCGCATGGAGCTGGAATACCACCAATACCAATATGGCAGCCCTGACCGATCCGCAGAATAAGATTGTGTATGAAATGCATCAGTACCTGGATAGCGATAGTAGCGGTACCCATGCCGAATGTGTGAGTAGTACCATTGGCGCACAGCGCGTTGTTGGCGCCACCCAGTGGCTGCGCGCAAATGGTAAACTGGGTGTTCTGGGCGAATTTGCCGGCGGTGCAAATGCCGTGTGTCAGCAGGCAGTTACCGGCCTGCTGGATCATCTGCAGGATAATAGTGATGTTTGGCTGGGCGCACTGTGGTGGGCAGCCGGTCCTTGGTGGGGCGATTATATGTATAGCTTTGAACCGCCGAGCGGCACCGGTTATGTTAATTATAATAGCATTCTGAAGAAGTACCTGCCG |
| Cel5A_39 | GGTCCTTGGCAGCAGTGTGGTGGCATTGGCTGGCAGGGCAGTACCGATTGTGTGAGCGGCTATCATTGCGTGTATCAGAATGATTGGTATAGTCAGTGCGTGCCGGGTGCCGCAAGCACCACCCTGCAGACCAGCACCACCAGCCGTCCGACCGCCACCAGTACCGCCCCTCCTAGCAGCACCACCAGTCCGAGTAAAGGCAAACTGAAATGGCTGGGTAGTAATGAAAGCGGTGCCGAATTTGGTGAAGGTAATTATCCGGGTCTGTGGGGTAAACATTTTACCTTTCCGAGTACCAGTACCATTCAGACCCTGATTAATGATGGCTATAATATTTTCCGCATCGATTTTAGCATGGAACGTCTGGTGCCGAATCAGCTGACCAGTAGCTTTGATCAGGGCTATCTGCGCAATCTGACCGAAGTTGTTAATTTTGTTACCAATGCAGGCGCCTATGCAGTTCTGGATCCGCATAATTATGGTCGCTATTATGGCAATATTATCACCGATACCAATGCCTTTCGCACCTTTTGGACCAATCTGGCCAAACAGTTTGCCAGTAATAGTCTGGTTATTTTTGATACCAACAACGAATATAACAGCATGGATCAGACCCTGGTGCTGAATCTGAATCAGGCAGCCATTAATGGCATTCGTGCAGCAGGCGCCACCAGTCAGTATATTTTTGTTGAAGGTAATGCATGGAGTGGCGCCTGGAGTTGGAATACCACCAATACCAATATGGCCGCACTGACCGATCCGCAGAATAAGATTGTGTATGAAATGCATCAGTACCTGGATAGCGATAGTAGCGGTACCCATGCAGAATGTGTTAGTAGCACCATTGGTGCCCAGCGCGTTGTTGGTGCAACCCAGTGGCTGCGCGCAAATGGTAAACTGGGCGTTCTGGGCGAATTTGCCGGTGGTGCAAATGCCGTGTGCCAGCAGGCAGTTACCGGCCTGCTGGATCATCTGCAGAGCAATAGTGATGTTTGGCTGGGTGCACTGTGGTGGGCCGCCGGTCCTTGGTGGGGTGACTATATGTATAGTTTTGAACCGCCGAGTGGCACCGGTTATGTGAATTATAATAGCATTCTGAAGAAGTACCTGCCG |
| Cel5A_22 | GGTCCTTGGCAGCAGTGCGGTGGTATTGGTTGGCAGGGCCCGACCGATTGTGTTAGTGGCTATCATTGTGTTTATCAGAATGATTGGTACAGCCAGTGCGTGCCGGGCGCAGCAAGCACCACCCTGCAGACCAGTACCACCAGTCGCCCGACCGCCACCAGCACCGCTCCTCCTAGCAGCACCACCAGTCCGCCGAAAGGTAAACTGAAATGGCTGGGCAGTAATGAAAGCGGTGCAGAATTTGGTGAAGGTAATTATCCGGGCCTGTGGGGCAAAGATTTTATTTTTCCGAGTACCAGCGCCATTCAGACCCTGATTAATGATGGCTATAATATTTTCCGTATCGATTTTAGCATGGAACGCCTGGTGCCGAATCAGCTGACCAGCAGTTTTGATCAGGGCTATCTGCGTAATCTGACCGAAGTGGTGAATTTTGTGACCAATGCCGGCGCCTATGCCGTTCTGGATCCGCATAATTATGGTCGTTATTATGGTAATATCATCACCGATACCAATGCATTTCGTACCTTTTGGACCAATCTGGCCAAACAGTTTGCCAGTAATAGCCTGGTTATTTTTGATACCAATAACGAATACAACACCATGGATCAGACCCTGGTTCTGAATCTGAATCAGGCAGCAATTGATGGTATTCGCGCCGCCGGCGCAACCAGTCAGTATATTTTTGTGGAAGGCAATGCCTGGAGCGGCGCCTGGAGTTGGAATACCACCAATACCAATATGGCCGCACTGACCGATCCGCAGAATAAGATTGTTTATGAAATGCATCAGTACCTGGATAGTGATAGCAGTGGTACCCATGCAGAATGCGTTAGTAGCACCATTGGCGCACAGCGTGTGGTGGGTGCCACCCAGTGGCTGCGCGCAAATGGTAAACTGGGTGTTCTGGGCGAATTTGCAGGTGGTGCCAATGCCGTTTGTCAGCAGGCAGTGACCGGCCTGCTGGATCATCTGCAGGATAATAGTGATGTGTGGCTGGGTGCACTGTGGTGGGCAGCAGGCCCGTGGTGGGGTACCTATATGTATAGTTTTGAACCGCCGAGCGGTACCGGCTATACCAATTATAATAGTATTCTGAAGAAGTACGCCCCG |
| Cel5A_79 | GGTCCTTGGCAGCAGTGTGGCGGCATTGGTTGGCAGGGTAGCACCGATTGCGTGGCACCGTATACCTGTGTTTATCAGAATGATTGGTATAGCCAGTGTGTGCCGGGTGCCGCAAGTACCACCCTGCAGACCAGCACCACCAGCGCACCGACCGCCACCAGCACCGCACCTCCTAGTAGCACCACCTCACCGAGTAAAGGCAAACTGAAATGGCTGGGCAGCAATGAAAGCGGCGCCGAATTTGGTGAAGGCAATTATCCGGGTCTGTGGGGCAAACATTTTATTTTTCCGAGTACCAGTGCCATTCAGACCCTGATTAATGATGGTTATAATATTTTCCGCATCGATTTTAGCATGGAACGTCTGGTTCCGAATCAGCTGACCAGTAGCTTTGATCAGGGCTATCTGCGCAATCTGACCGAAGTTGTTAATTTTGTGACCAATGCCGGCAAATATGCCGTTCTGGATCCGCATAATTATGGTCGCTATTATGGTAATATCATCACCGATACCAATGCCTTTCGTACCTTTTGGACCAATCTGGCAAAACAGTTTGCAAGCAATAGCCTGGTTATTTTTGATACCAATAACGAATACAACACCATGGATCAGACCCTGGTTCTGAATCTGAATCAGGCCGCAATTGATGGTATTCGTGCCGCCGGCGCCACCAGCCAGTATATTTTTGTGGAAGGTAATGCATGGAGCGGTGCCTGGAGTTGGAATACCACCAATACCAATATGGCCGCCCTGACCGATCCGCAGAATAAGATTGTTTATGAAATGCATCAGTACCTGGATAGCGATAGTAGTGGTACCCATGCAGAATGCGTTAGCAGTACCATTGGTGCCCAGCGTGTTGTGGGTGCAACCCAGTGGCTGCGCGCAAATAATAAGCTGGGCGTTCTGGGCGAATTTGCCGGTGGCGCCAATGCAGTGTGCCAGCAGGCAGTTACCGGCCTGCTGGATTATATGCAGGATAATAGTGATGTTTGGCTGGGCGCCCTGTGGTGGGCAGCAGGTCCTTGGTGGGGCGATTATATGTATAGCTTTGAACCGCCGAGTGGTACCGGCTATGTGAATTATAATAGCATTCTGAAAAAGTACCTGCCG |

## Table S9 The amino acid sequences of I6M and its three mutants

| Protein | Sequence |
| --- | --- |
| I6M | SNPYQRGPNPTRSALTATGPFSVATYTVSRLLVSGFGGGVIYYPTGTSLTFGGIAMSPGYTADASSLAWLGRRLASHGFVVLVINTNSRFDGPDSRARQLSAALNYLRTSSPSAVRARLDANRLAVAGHSMGGGGTLRIAEQNPSLKAAVPLTPWHPDKTFNTSVPVLIVGAQADTVAPVSQHAIPFYQNLPSTTPKVYVELCNASHIAPNSPNAAISVYTISWMKLWVDNDTRYRQFLCNVNDPALCDFRTNNRHCQ |
| I6M_75 | SNPYQRGPDPTRSALTATGPFSVATYTVSRLLVSGFGGGTIYYPTGTSLTFGGIAMSPGYTADASSLAWLGRRLASHGFVVLVINTNSRFDGPDSRARQLSAALNYLRTSSPSAVRARIDANRLAVAGHSMGGGGTLRIAEQNPSLKAAVPLTPWNPDKTFNTSTPVLIVGAQADTVAPVSQHAIPFYQNLPSTTPKVYVELCNASHIAPNSPNTAISVYTISWMKLWVDNDTRYRQFLCNVNDPALCDFRTNNPHCQ |
| I6M_69 | SNPYQRGPNPTRAALTATGPFSVATYTVSRLLVSGFGGGVIYYPTGTNLTFGGIAMAPGYTADASSLAWLGPRLASHGFVVLVIDTNSRFDGPDSRARQLSAALNYLRTSSPSAVRARLDPNRLAVAGHSMGGGGTLRIAEQNPSLKAAVPLTPWHPDKTFNTRVPVLIVGAQADTVAPVSQHAIPFYQNLPSTTPKVYVELCNASHIAPNSPNATISVYGISWMKLWVDNDTRYRQFLCNVNDPALCDFRTNNRHCQ |
| I6M_10 | SNPYQRGPNPTRAALTATGPFSVATYTVSRLLVSGFGGGTIYYPTGTSLTFGGIAMSPGYTADQSSLAWLGRRLASHGFVVLVIDTNSRYDGPDSRARQLSAALNYLRTSSPSAVRARLDANRLAVAGHSMGGGGTLRIAEQNPSLKAAVPLTPWNPDKTFNTSTPVLIVGAQADTVAPVSQHAIPFYNNLPSTTPKVYVELCNASHIAPNSPNAAIAVYTISWLKLWVDNDTRYRQFLCNVNDPALCDFRTNNRHCQ |

## Table S10 I6M and its three mutant *E. coli* codon-optimized DNA sequences

| Protein | Sequence |
| --- | --- |
| I6M | TCAAATCCTTATCAAAGGGGACCCAACCCAACTCGCAGCGCACTGACGGCGACCGGCCCATTCAGCGTTGCGACCTATACGGTGTCCAGATTGCTGGTAAGCGGTTTCGGCGGCGGTGTTATTTATTACCCGACCGGTACATCGTTGACTTTCGGTGGCATCGCTATGTCCCCGGGCTACACCGCCGACGCATCAAGCTTGGCCTGGCTGGGTCGTCGTCTGGCTTCGCACGGCTTCGTGGTGTTGGTTATCAACACCAACAGCCGCTTTGATGGTCCGGATAGCCGTGCGCGCCAACTGTCTGCCGCGCTGAATTACCTGCGCACCAGTAGCCCGTCCGCGGTGCGTGCCCGTCTGGACGCGAATCGTCTGGCGGTCGCGGGTCACAGCATGGGTGGTGGCGGCACGCTGCGCATCGCCGAGCAGAACCCGAGCCTTAAGGCAGCGGTTCCGCTGACCCCGTGGCACCCGGATAAGACCTTCAACACCAGCGTGCCGGTTCTGATCGTGGGTGCGCAGGCAGACACCGTGGCTCCGGTGAGCCAACATGCGATTCCGTTTTATCAGAACCTCCCTTCGACCACCCCAAAAGTTTACGTTGAACTGTGCAATGCGTCCCATATTGCCCCGAATTCCCCGAACGCTGCGATTAGCGTCTACACGATCTCTTGGATGAAACTGTGGGTTGATAACGACACCCGTTATCGTCAGTTTTTGTGCAACGTCAATGACCCGGCATTATGCGATTTTCGTACCAATAACCGCCACTGTCAA |
| I6M_75 | AGTAACCCGTATCAGCGCGGCCCGGATCCGACCCGTAGCGCATTAACCGCAACCGGCCCGTTTAGCGTTGCAACCTATACCGTTAGCCGCCTGCTGGTGAGCGGTTTTGGCGGTGGTACCATCTATTATCCGACCGGTACCAGTCTGACCTTTGGCGGCATTGCCATGAGCCCGGGCTATACCGCAGATGCCAGCAGTCTGGCCTGGCTGGGCCGTCGTCTGGCAAGCCATGGTTTTGTGGTTCTGGTTATTAATACCAATAGCCGTTTTGATGGTCCGGATAGCCGTGCCCGTCAGCTGAGCGCCGCACTGAATTATCTGCGCACCAGTAGTCCGAGTGCCGTGCGTGCACGTATTGATGCAAATCGTCTGGCAGTTGCAGGCCATAGCATGGGTGGTGGTGGTACCCTGCGTATTGCAGAACAGAATCCGAGTCTGAAAGCCGCCGTGCCGCTGACCCCGTGGAATCCTGATAAAACCTTTAATACCAGTACCCCGGTTCTGATTGTTGGCGCACAGGCAGATACCGTTGCACCGGTGAGTCAGCATGCAATTCCGTTTTATCAGAATCTGCCGAGTACCACCCCGAAAGTTTATGTTGAACTGTGTAATGCAAGTCATATTGCACCGAATAGTCCGAATACCGCAATTAGTGTTTATACCATTAGTTGGATGAAGCTGTGGGTGGATAATGATACCCGCTATCGCCAGTTTCTGTGTAATGTGAATGATCCGGCACTGTGCGATTTTCGCACCAATAATCCGCATTGCCAG |
| I6M_69 | AGTAACCCGTATCAGCGCGGCCCGAATCCGACCCGTGCAGCTCTGACCGCCACCGGTCCGTTTAGTGTGGCCACCTATACCGTGAGTCGTCTGCTGGTTAGTGGTTTTGGTGGTGGCGTTATCTATTATCCGACCGGTACCAATCTGACCTTTGGTGGCATTGCCATGGCACCGGGCTATACCGCAGATGCAAGTAGCCTGGCATGGCTGGGTCCGCGTCTGGCCAGTCATGGCTTTGTTGTTCTGGTTATTGATACCAATAGCCGCTTTGATGGTCCGGATAGTCGTGCCCGCCAGCTGAGTGCAGCCCTGAATTATCTGCGTACCAGCAGCCCGAGCGCAGTTCGCGCCCGTCTGGATCCGAATCGTCTGGCAGTGGCAGGCCATAGTATGGGCGGCGGCGGTACCCTGCGTATTGCAGAACAGAATCCGAGCCTGAAAGCCGCAGTTCCGCTGACCCCGTGGCATCCGGATAAAACCTTTAATACCCGCGTGCCGGTGCTGATTGTGGGCGCCCAGGCCGATACCGTGGCACCTGTGAGTCAGCATGCCATTCCGTTTTATCAGAATCTGCCGAGCACCACCCCGAAAGTTTATGTGGAACTGTGCAATGCCAGCCATATTGCCCCGAATAGTCCGAATGCCACCATTAGCGTGTATGGCATTAGCTGGATGAAACTGTGGGTTGATAATGATACCCGTTATCGCCAGTTTCTGTGTAATGTTAATGATCCGGCACTGTGTGATTTTCGCACCAATAATCGTCATTGCCAG |
| I6M_10 | AGCAATCCGTATCAGCGTGGTCCGAATCCGACCCGTGCCGCACTGACCGCCACCGGTCCTTTTAGCGTTGCAACCTATACCGTGAGCCGTCTGCTGGTGAGTGGCTTTGGTGGCGGTACCATCTATTATCCGACCGGTACCAGCCTGACCTTTGGTGGCATTGCCATGAGCCCGGGCTATACCGCCGATCAGAGCAGCCTGGCATGGCTGGGCCGTCGTCTGGCAAGCCATGGCTTTGTTGTTCTGGTTATTGATACCAATAGCCGCTATGATGGTCCGGATAGCCGTGCACGCCAGCTGAGTGCCGCACTGAATTATCTGCGTACCAGCAGCCCGAGCGCAGTTCGCGCCCGTCTGGATGCAAATCGTCTGGCAGTGGCAGGTCATAGCATGGGCGGCGGCGGTACCCTGCGTATTGCAGAACAGAATCCGAGTCTGAAAGCCGCCGTTCCGCTGACCCCGTGGAATCCGGATAAAACCTTTAATACCAGTACCCCGGTTCTGATTGTGGGTGCACAGGCAGATACCGTTGCACCGGTTAGCCAGCATGCAATTCCGTTTTATAATAATCTGCCGAGTACCACCCCGAAAGTTTATGTGGAACTGTGTAATGCAAGTCATATTGCCCCGAATAGCCCGAATGCCGCCATTGCCGTGTATACCATTAGTTGGCTGAAACTGTGGGTTGATAATGATACCCGTTATCGTCAGTTTCTGTGTAATGTTAATGATCCGGCCCTGTGTGATTTTCGCACCAATAATCGTCATTGTCAG |

## Table S11 Attention values of various parts of xylanase Pm10868 and its mutants

| Protein | First shell | Second shell | Not in shells | Activate site | CBM | GH10 domain |
| --- | --- | --- | --- | --- | --- | --- |
| Pm10868 | 1.100E-03 | 1.112E-03 | 1.399E-03 | 8.820E-04 | 1.680E-03 | 1.230E-03 |
| Pm10868_85 | 1.154E-03 | 1.121E-03 | 1.403E-03 | 9.070E-04 | 1.693E-03 | 1.233E-03 |
| Pm10868_11 | 1.063E-03 | 1.106E-03 | 1.399E-03 | 8.545E-04 | 1.718E-03 | 1.212E-03 |
| Pm10868_04 | 1.126E-03 | 1.135E-03 | 1.404E-03 | 8.620E-04 | 1.665E-03 | 1.249E-03 |

## Table S12 Amino acid biosynthetic cost

| Amino acid | Cost | Amino acid | Cost | Amino acid | Cost | Amino acid | Cost |
| --- | --- | --- | --- | --- | --- | --- | --- |
| A | 14.5 | G | 14.5 | M | 36.5 | S | 14.5 |
| C | 26.5 | H | 29.0 | N | 18.5 | T | 21.5 |
| D | 15.5 | I | 38.0 | P | 14.5 | V | 29.0 |
| E | 9.5 | K | 36.0 | Q | 10.5 | W | 75.5 |
| F | 61.0 | L | 37.0 | R | 20.5 | Y | 59.0 |

## Table S13 Mean evaluation results of MPB-EXP-R models for each species on independent test sets

| taxon ID | Species or straintaxon ID Species or strain | rmse | r2 | pearson_r | | pearson_p | | spearman_r | spearman_p |
| --- | --- | --- | --- | --- | --- | --- | --- | --- | --- |
| 547559 | *Natrialba magadii* ATCC 43099 | 0.687 | 0.181 | | 0.429 | | 5.74E-18 | 0.438 | 1.03E-18 |
| 64091 | *Halobacterium salinarum* NRC-1 | 0.687 | 0.455 | | 0.675 | | 1.19E-47 | 0.673 | 3.45E-47 |
| 880073 | *Caldithrix abyssi* DSM 13497 | 0.618 | 0.385 | | 0.626 | | 1.16E-46 | 0.624 | 2.68E-46 |
| 189518 | *Leptospira interrogans* serovar Lai str. 56601 | 0.652 | 0.339 | | 0.602 | | 1.47E-48 | 0.605 | 5.29E-49 |
| 246200 | *Ruegeria pomeroyi* DSS-3 | 0.532 | 0.333 | | 0.58 | | 6.32E-37 | 0.571 | 1.14E-35 |
| 208964 | *Pseudomonas aeruginosa* PAO1 | 1.067 | 0.362 | | 0.602 | | 4.44E-82 | 0.608 | 4.52E-84 |
| 160488 | *Pseudomonas putida* KT2440 | 0.498 | 0.399 | | 0.635 | | 2.30E-47 | 0.62 | 9.03E-45 |
| 99287 | *Salmonella enterica* subsp. enterica serovar Typhimurium str. LT2 | 0.673 | 0.33 | | 0.577 | | 2.78E-47 | 0.565 | 6.34E-45 |
| 511145 | *Escherichia coli* str. K-12 substr. MG1655 | 0.989 | 0.404 | | 0.636 | | 1.32E-84 | 0.631 | 9.97E-83 |
| 272620 | *Klebsiella pneumoniae* subsp. pneumoniae MGH 78578 | 0.75 | 0.325 | | 0.575 | | 4.53E-42 | 0.571 | 1.86E-41 |
| 1286170 | *Raoultella ornithinolytica* B6 | 0.759 | 0.199 | | 0.454 | | 5.16E-26 | 0.469 | 6.45E-28 |
| 709991 | *Odoribacter splanchnicus* DSM 20712 | 0.702 | 0.462 | | 0.691 | | 3.70E-59 | 0.67 | 1.58E-54 |
| 537011 | *Segatella copri* DSM 18205 | 0.643 | 0.44 | | 0.672 | | 1.00E-45 | 0.652 | 3.28E-42 |
| 411477 | *Parabacteroides merdae* ATCC 43184 | 0.648 | 0.505 | | 0.71 | | 6.37E-66 | 0.708 | 2.31E-65 |
| 435591 | *Parabacteroides distasonis* ATCC 8503 | 0.674 | 0.404 | | 0.64 | | 3.13E-47 | 0.622 | 6.32E-44 |
| 435590 | *Phocaeicola vulgatus* ATCC 8482 | 0.698 | 0.424 | | 0.658 | | 1.02E-52 | 0.642 | 1.50E-49 |
| 226186 | *Bacteroides thetaiotaomicron* VPI-5482 | 0.718 | 0.456 | | 0.679 | | 1.59E-63 | 0.664 | 6.05E-60 |
| 272559 | *Bacteroides fragilis* NCTC 9343 | 0.694 | 0.417 | | 0.653 | | 1.02E-52 | 0.626 | 2.22E-47 |
| 411479 | *Bacteroides uniformis* ATCC 8492 | 0.657 | 0.467 | | 0.686 | | 1.03E-61 | 0.678 | 8.55E-60 |
| 449447 | *Microcystis aeruginosa* NIES-843 | 0.78 | 0.255 | | 0.511 | | 1.75E-57 | 0.439 | 4.67E-41 |
| 1140 | *Synechococcus elongatus* PCC 7942 = FACHB-805 | 0.582 | 0.593 | | 0.77 | | 1.50E-86 | 0.76 | 4.84E-83 |
| 100226 | *Streptomyces coelicolor* A3(2) | 0.08 | -0.038 | | 0.359 | | 2.10E-22 | 0.401 | 4.67E-28 |
| 83332 | *Mycobacterium tuberculosis* H37Rv | 0.733 | 0.411 | | 0.642 | | 4.56E-79 | 0.639 | 4.46E-78 |
| 246196 | *Mycolicibacterium smegmatis* MC2 155 | 0.752 | 0.342 | | 0.594 | | 1.90E-56 | 0.588 | 5.57E-55 |
| 1280 | *Staphylococcus aureus* | 0.607 | 0.458 | | 0.678 | | 6.53E-47 | 0.671 | 8.91E-46 |
| 224308 | *Bacillus subtilis* subsp. subtilis str. 168 | 0.755 | 0.43 | | 0.657 | | 3.68E-100 | 0.628 | 5.81E-89 |
| 610130 | *[Clostridium] saccharolyticum* WM1 | 0.68 | 0.378 | | 0.617 | | 2.34E-45 | 0.573 | 7.38E-38 |
| 411902 | *Enterocloster bolteae* ATCC BAA-613 | 0.719 | 0.357 | | 0.606 | | 1.13E-43 | 0.568 | 1.54E-37 |
| 411470 | *[Ruminococcus] gnavus* ATCC 29149 | 0.64 | 0.447 | | 0.671 | | 4.27E-42 | 0.64 | 2.53E-37 |
| *55529* | *Guillardia theta* | 0.667 | 0.542 | | 0.738 | | 1.30E-303 | 0.702 | 3.24E-262 |
| 44689 | *Dictyostelium discoideum* | 0.704 | 0.538 | | 0.737 | | 4.16E-233 | 0.724 | 7.92E-221 |
| 2903 | *Emiliania huxleyi* | 0.623 | 0.503 | | 0.709 | | 1.31E-235 | 0.679 | 2.20E-208 |
| 5691 | *Trypanosoma brucei* | 0.934 | 0.396 | | 0.635 | | 2.33E-121 | 0.626 | 9.25E-117 |
| 353153 | *Trypanosoma cruzi strain CL Brener* | 0.963 | 0.417 | | 0.653 | | 1.15E-27 | 0.62 | 2.72E-24 |
| 2850 | *Phaeodactylum tricornutum* | 0.645 | 0.538 | | 0.736 | | 1.00E-186 | 0.725 | 1.12E-178 |
| 35128 | *Thalassiosira pseudonana* | 0.666 | 0.472 | | 0.69 | | 2.99E-191 | 0.676 | 2.02E-181 |
| 5811 | *Toxoplasma gondii* | 0.768 | 0.413 | | 0.655 | | 4.06E-62 | 0.646 | 8.12E-60 |
| 5833 | *Plasmodium falciparum* | 0.803 | 0.513 | | 0.722 | | 2.42E-162 | 0.673 | 2.17E-133 |
| 73239 | *Plasmodium yoelii yoelii* | 0.588 | 0.362 | | 0.629 | | 4.99E-35 | 0.618 | 1.46E-33 |
| 3055 | *Chlamydomonas reinhardtii* | 0.787 | 0.482 | | 0.695 | | 7.29E-186 | 0.679 | 2.60E-174 |
| 3218 | *Physcomitrium patens* | 0.744 | 0.509 | | 0.718 | | 1.25E-260 | 0.708 | 4.36E-250 |
| 4577 | *Zea mays* | 0.839 | 0.402 | | 0.637 | | 1.22E-234 | 0.614 | 5.01E-214 |
| 39947 | *Oryza sativa Japonica Group* | 0.767 | 0.461 | | 0.682 | | 4.02E-255 | 0.672 | 4.28E-245 |
| 4565 | *Triticum aestivum* | 1.31 | 0.069 | | 0.28 | | 1.76E-12 | 0.288 | 3.70E-13 |
| 4513 | *Hordeum vulgare* | 0.827 | 0.351 | | 0.604 | | 4.84E-60 | 0.592 | 5.53E-57 |
| 4097 | *Nicotiana tabacum* | 0.728 | 0.461 | | 0.683 | | 1.95E-40 | 0.654 | 5.18E-36 |
| 4081 | *Solanum lycopersicum* | 0.348 | 0.189 | | 0.437 | | 3.57E-69 | 0.337 | 4.37E-40 |
| 4113 | *Solanum tuberosum* | 1.027 | 0.522 | | 0.723 | | 5.82E-195 | 0.71 | 6.32E-185 |
| 29760 | *Vitis vinifera* | 0.39 | 0.441 | | 0.667 | | 3.94E-53 | 0.659 | 1.94E-51 |
| 3880 | *Medicago truncatula* | 0.823 | 0.489 | | 0.705 | | 0.00E+00 | 0.682 | 1.52e-319 |
| 3847 | *Glycine max* | 0.897 | 0.394 | | 0.628 | | 1.60E-29 | 0.519 | 5.09E-19 |
| 2711 | *Citrus sinensis* | 0.756 | 0.397 | | 0.632 | | 4.42E-59 | 0.619 | 4.99E-56 |
| 3635 | *Gossypium hirsutum* | 0.951 | 0.318 | | 0.564 | | 1.37E-69 | 0.554 | 1.87E-66 |
| 3702 | *Arabidopsis thaliana* | 0.847 | 0.56 | | 0.755 | | 0.00E+00 | 0.752 | 0.00E+00 |
| 3708 | *Brassica napus* | 0.601 | 0.41 | | 0.647 | | 6.08E-64 | 0.629 | 1.98E-59 |
| 214684 | *Cryptococcus neoformans* var. neoformans JEC21 | 0.878 | 0.41 | | 0.646 | | 4.24E-58 | 0.644 | 1.46E-57 |
| 4896 | *Schizosaccharomyces pombe* | 0.693 | 0.385 | | 0.629 | | 5.05E-100 | 0.665 | 8.37E-116 |
| 5507 | *Fusarium oxysporum* | 0.738 | 0.387 | | 0.626 | | 9.06E-93 | 0.599 | 2.98E-83 |
| 5141 | *Neurospora crassa* | 0.661 | 0.508 | | 0.724 | | 1.21E-167 | 0.718 | 1.74E-163 |
| 5476 | *Candida albicans* | 0.684 | 0.511 | | 0.721 | | 2.12E-124 | 0.718 | 1.29E-122 |
| 284590 | *Kluyveromyces lactis* NRRL Y-1140 | 0.45 | 0.541 | | 0.74 | | 1.31E-101 | 0.718 | 4.23E-93 |
| 4932 | *Saccharomyces cerevisiae* | 0.546 | 0.638 | | 0.799 | | 1.13E-266 | 0.794 | 2.56E-261 |
| 6239 | *Caenorhabditis elegans* | 0.829 | 0.525 | | 0.729 | | 0.00E+00 | 0.723 | 0.00E+00 |
| 6945 | *Ixodes scapularis* | 0.312 | 0.2 | | 0.467 | | 5.33E-43 | 0.45 | 1.13E-39 |
| 7460 | *Apis mellifera* | 0.972 | 0.449 | | 0.672 | | 5.95E-89 | 0.659 | 2.48E-84 |
| 7227 | *Drosophila melanogaster* | 0.748 | 0.516 | | 0.722 | | 0.00E+00 | 0.697 | 0.00E+00 |
| 7165 | *Anopheles gambiae* | 0.848 | 0.421 | | 0.649 | | 5.45E-96 | 0.633 | 8.82E-90 |
| 7159 | *Aedes aegypti* | 0.814 | 0.368 | | 0.612 | | 1.48E-75 | 0.597 | 5.58E-71 |
| 7955 | *Danio rerio* | 1.016 | 0.545 | | 0.74 | | 0.00E+00 | 0.747 | 0.00E+00 |
| 30732 | *Oryzias melastigma* | 0.707 | 0.455 | | 0.676 | | 9.11E-165 | 0.659 | 1.11E-153 |
| 8030 | *Salmo salar* | 0.843 | 0.429 | | 0.667 | | 8.67E-40 | 0.605 | 3.51E-31 |
| 8022 | *Oncorhynchus mykiss* | 0.791 | 0.392 | | 0.627 | | 1.46E-76 | 0.597 | 6.02E-68 |
| 8364 | *Xenopus tropicalis* | 0.75 | 0.26 | | 0.55 | | 2.00E-29 | 0.573 | 2.57E-32 |
| 8355 | *Xenopus laevis* | 1.096 | 0.405 | | 0.64 | | 1.81E-54 | 0.629 | 4.01E-52 |
| 9031 | *Gallus gallus* | 0.872 | 0.477 | | 0.694 | | 0.00E+00 | 0.653 | 6.52E-288 |
| 9612 | *Canis lupus* | 0.724 | 0.44 | | 0.666 | | 3.91E-184 | 0.653 | 9.11E-175 |
| 9615 | *Canis lupus(familiaris)* | 0.895 | 0.427 | | 0.659 | | 9.98E-95 | 0.676 | 3.25E-101 |
| 9796 | *Equus caballus* | 0.781 | 0.337 | | 0.591 | | 8.39E-55 | 0.566 | 1.57E-49 |
| 9823 | *Sus scrofa* | 0.87 | 0.443 | | 0.666 | | 6.56E-180 | 0.635 | 3.62E-158 |
| 9913 | *Bos taurus* | 1.175 | 0.36 | | 0.602 | | 1.29E-201 | 0.612 | 4.72E-210 |
| 89462 | *Bubalus bubalis* | 0.711 | 0.297 | | 0.547 | | 1.68E-35 | 0.539 | 2.17E-34 |
| 9544 | *Macaca mulatta* | 0.935 | 0.453 | | 0.674 | | 4.55E-223 | 0.532 | 2.68E-123 |
| 9598 | *Pan troglodytes* | 0.544 | 0.358 | | 0.598 | | 1.40E-56 | 0.595 | 9.85E-56 |
| 9606 | *Homo sapiens* | 0.938 | 0.511 | | 0.719 | | 0.00E+00 | 0.722 | 0.00E+00 |
| 9986 | *Oryctolagus cuniculus* | 0.552 | 0.39 | | 0.628 | | 5.75E-115 | 0.595 | 3.08E-100 |
| 10029 | *Cricetulus griseus* | 0.755 | 0.387 | | 0.625 | | 8.04E-128 | 0.617 | 4.74E-124 |
| 10090 | *Mus musculus* | 1.128 | 0.515 | | 0.72 | | 0.00E+00 | 0.731 | 0.00E+00 |
| 10116 | *Rattus norvegicus* | 0.955 | 0.452 | | 0.675 | | 0.00E+00 | 0.679 | 0.00E+00 |

## Table S14 Xylose solution with different concentration gradient at OD_540_

| No. | 1 | 2 | 3 | 4 | 5 | 6 | 7 | 8 | 9 | 10 |
| --- | --- | --- | --- | --- | --- | --- | --- | --- | --- | --- |
| OD1 | 0.110 | 0.295 | 0.530 | 0.760 | 1.032 | 1.123 | 1.282 | 1.526 | 1.635 | 1.776 |
| OD2 | 0.107 | 0.292 | 0.527 | 0.780 | 1.063 | 1.091 | 1.273 | 1.532 | 1.636 | 1.758 |
| OD3 | 0.108 | 0.291 | 0.542 | 0.791 | 1.082 | 1.13 | 1.283 | 1.544 | 1.647 | 1.756 |
| OD | 0.108 | 0.292 | 0.533 | 0.777 | 1.059 | 1.115 | 1.279 | 1.534 | 1.639 | 1.763 |
| ΔOD | 0.000 | 0.184 | 0.425 | 0.669 | 0.951 | 1.006 | 1.171 | 1.426 | 1.531 | 1.655 |

# Supplementary References

[1] W. Li, A. Godzik, *Bioinformatics* **2006**, *22* (13), 1658, <https://doi.org/10.1093/bioinformatics/btl158>.

[2] C. Vogel, R. de Sousa Abreu, D. Ko, S. Y. Le, B. A. Shapiro, S. C. Burns, D. Sandhu, D. R. Boutz, E. M. Marcotte, L. O. Penalva, *Molecular systems biology* **2010**, *6* (1), 400.

[3] D. Szklarczyk, A. L. Gable, K. C. Nastou, D. Lyon, R. Kirsch, S. Pyysalo, N. T. Doncheva, M. Legeay, T. Fang, P. Bork, *Nucleic Acids Research* **2021**, *49* (D1), D605, <https://doi.org/10.1093/nar/gkaa1074>.

[4] J. Devlin, M.-W. Chang, K. Lee, K. Toutanova, *ArXiv Preprint arXiv:1810.04805* **2018**.

[5] B. E. Suzek, Y. Wang, H. Huang, P. B. McGarvey, C. H. Wu, U. Consortium, *Bioinformatics* **2015**, *31* (6), 926, <https://doi.org/10.1093/bioinformatics/btu739>.

[6] Z. Ding, F. Guan, G. Xu, Y. Wang, Y. Yan, W. Zhang, N. Wu, B. Yao, H. Huang, T. Tuller, *Computational and Structural Biotechnology Journal* **2022**, *20*, 1142, <https://doi.org/10.1016/j.csbj.2022.02.030>.

[7] a) G. Gallo-Oller, R. Ordonez, J. Dotor, *Journal of immunological methods* **2018**, *457*, 1; b) M. Gassmann, B. Grenacher, B. Rohde, J. Vogel, *Electrophoresis* **2009**, *30* (11), 1845.

[8] M. D. Abràmoff, P. J. Magalhães, S. J. Ram, *Biophotonics international* **2004**, *11* (7), 36.
